# Prostaglandins and calprotectin are genetically and functionally linked to the Inflammatory Bowel Diseases

**DOI:** 10.1371/journal.pgen.1010189

**Published:** 2022-09-26

**Authors:** Mohamad Karaky, Gabrielle Boucher, Saraï Mola, Sylvain Foisy, Claudine Beauchamp, Marie-Eve Rivard, Melanie Burnette, Hugues Gosselin, iGenoMed Consortium, Alain Bitton, Guy Charron, Philippe Goyette, John D. Rioux

**Affiliations:** 1 Montreal Heart Institute Research Center, Montreal, Quebec, Canada; 2 A complete list of members and their affiliations can be found at the end of the manuscript; 3 McGill University Health Centre, Division of Gastroenterology, Montreal, Quebec, Canada; 4 Université de Montréal, Faculty of Medicine, Montreal, Quebec, Canada; New York Genome Center & Columbia University, UNITED STATES

## Abstract

**Background:**

Genome wide association studies (GWAS) have identified and validated more than 200 genomic loci associated with the inflammatory bowel disease (IBD), although for most the causal gene remains unknown. Given the importance of myeloid cells in IBD pathogenesis, the current study aimed to uncover the role of genes within IBD genetic loci that are endogenously expressed in this cell lineage.

**Methods:**

The open reading frames (ORF) of 42 genes from IBD-associated loci were expressed via lentiviral transfer in the THP-1 model of human monocytes and the impact of each of these on the cell’s transcriptome was analyzed using a RNA sequencing-based approach. We used a combination of genetic and pharmacologic approaches to validate our findings in the THP-1 line with further validation in human induced pluripotent stem cell (hiPSC)-derived-monocytes.

**Results:**

This functional genomics screen provided evidence that genes in four IBD GWAS loci (*PTGIR*, *ZBTB40*, *SLC39A11* and *NFKB1*) are involved in controlling *S100A8* and *S100A9* gene expression, which encode the two subunits of calprotectin (CP). We demonstrated that increasing PTGIR expression and/or stimulating *PTGIR* signaling resulted in increased CP expression in THP-1.

This was further validated in hiPSC-derived monocytes. Conversely, knocking-down PTGIR endogenous expression and/or inhibiting *PTGIR* signaling led to decreased CP expression. These analyses were extended to the known IBD gene *PTGER4*, whereby its specific agonist also led to increased CP expression. Furthermore, we demonstrated that the *PTGIR* and *PTGER4* mediated control of CP expression was dependent on signaling via adenylate cyclase and *STAT3*. Finally, we demonstrated that LPS-mediated increases in CP expression could be potentiated by agonists of *PTGIR* and *PTGER4*, and diminished by their antagonists.

**Conclusion:**

Our results support a causal role for the *PTGIR*, *PTGER4*, *ZBTB40*, *SLC39A11* and *NFKB1* genes in IBD, with all five genes regulating the expression of CP in myeloid cells, as well as potential roles for the prostacyclin/prostaglandin biogenesis and signaling pathways in IBD susceptibility and pathogenesis.

## Author summary

Crohn’s Disease and Ulcerative colitis are the two main types of inflammatory bowel diseases (IBD). These are debilitating chronic inflammatory diseases of the digestive tract. IBD pathogenesis is complex and involves multiple different cell types within the intestinal mucosa. While over 200 regions of the genome have been associated with susceptibility to IBD, for most the causal gene remains to be identified. In the current study we have focused on genes from IBD loci that are endogenously expressed in monocytes or macrophages, given the importance of these cells in IBD pathogenesis. Specifically, we modulated the expression of 42 genes from within validated IBD loci, in a human monocyte/macrophage cell line, and determined the impact of this increased expression on the rest of the transcriptome. We found evidence that four of these genes (*PTGIR*, *ZBTB40*, *SLC39A11* and *NFKB1*) control the expression of calprotectin, which is a proinflammatory molecule that is used as a marker of intestinal mucosal inflammation. We then elucidated how prostaglandin signaling via prostaglandin receptors PTGIR and PTGER4, another IBD gene, regulates calprotectin expression. This work provides evidence that all five genes are likely causal and provide the first link between calprotectin and disease susceptibility.

## Introduction

Inflammatory bowel diseases (IBD) are chronic inflammatory diseases of the digestive system. Crohn’s disease (CD) and ulcerative colitis (UC) are the two most common subtypes of IBD [1]. IBD is a global disease with increasing prevalence, estimated to reach four million patients in North America by 2030 [2]. While the etiology of IBD is still not fully known, there is growing evidence suggesting that there is a combination of genetic and environmental risk factors impacting on disease susceptibility, with the latter including gut microbial components that chronically stimulate the gastrointestinal immune system [3].To this point, it is clear that the innate and adaptive immune systems are both implicated in IBD pathology. For example, in the innate immune system, neutrophil infiltration and activation have been correlated with UC severity [4]. CD14^+^ CD16^+^ monocyte infiltrates in the inflamed mucosa have been identified as a major proinflammatory immune cell population in CD [5]. Furthermore, in IBD, macrophages massively infiltrate the intestinal mucosa [6] and are considered important effectors of pathology, producing inflammatory mediators such as TNF-alpha, IL1, IL6, and nitric oxide [7]. This is further supported by recent single cell RNA sequencing of intestinal tissues demonstrating an important enrichment of monocytes, inflammatory M1 macrophages, activated DCs and plasmacytoid DCs in inflamed tissues from various gut locations in IBD patients and has been correlated with disease severity, while in contrast, the anti-inflammatory M2 subset was diminished with severity [8].

In terms of genetic risk, > 200 genomic loci have been associated with IBD, CD or UC [9–11]. Interestingly, a transcriptome-based analysis of the genes located within these IBD loci found an enrichment of expression within various immune cells [12]. In addition, regulatory variants in IBD loci were active in different immune cells such as CD4+ T, CD8+ T, CD19+ B cells and CD14+ monocytes [13]. While genetic and subsequent functional studies have identified a handful of causal genes from these loci that are believed to act primarily within cells of the monocyte/macrophage lineage (e.g *IRGM*, *LRRK2*, *CARD9*), no large-scale functional screen of IBD genes had been performed in this cellular context. Given this, additional approaches are necessary to resolve ~95% of IBD loci. It is believed that most association signals at Genome Wide Association Studies (GWAS) loci could be explained by common regulatory variants that control the expression of one or more genes in disease relevant cell types [13,14].

Given these observations, we propose that expression-based screens of genes within IBD loci can provide valuable information regarding a gene’s function within a specific cellular context and functionally link different genes through their shared impacts on the cell’s transcriptome. In the current study we have focused on genes from IBD loci that are endogenously expressed in monocytes or macrophages, given the importance of these cells in IBD pathogenesis. Specifically, we modulated the expression of 42 genes from within validated IBD loci and determined the impact of this increased expression on the rest of the transcriptome. This functional genomics screen has provided evidence that genes in four IBD loci (*PTGIR*, *ZBTB40*, *SLC39A11* and *NFKB1*) are involved in controlling the expression levels of *S100A8* and *S100A9*, the two subunits of CP, an important IBD biomarker for monitoring disease severity [15,16]. Given this, we explored the effect of the prostaglandin (PG) receptors *PTGIR* and *PTGER4* associated with IBD [17], receptors of PG I2 and PG E2 respectively, on regulating the expression of CP in THP-1 cells and hiPSC-derived monocytes in order to elucidate the role of the PG pathway in IBD pathology.

## Results

### Functional genomics screen of 42 IBD genes in the human THP-1 monocytic line

**Fig 1:**
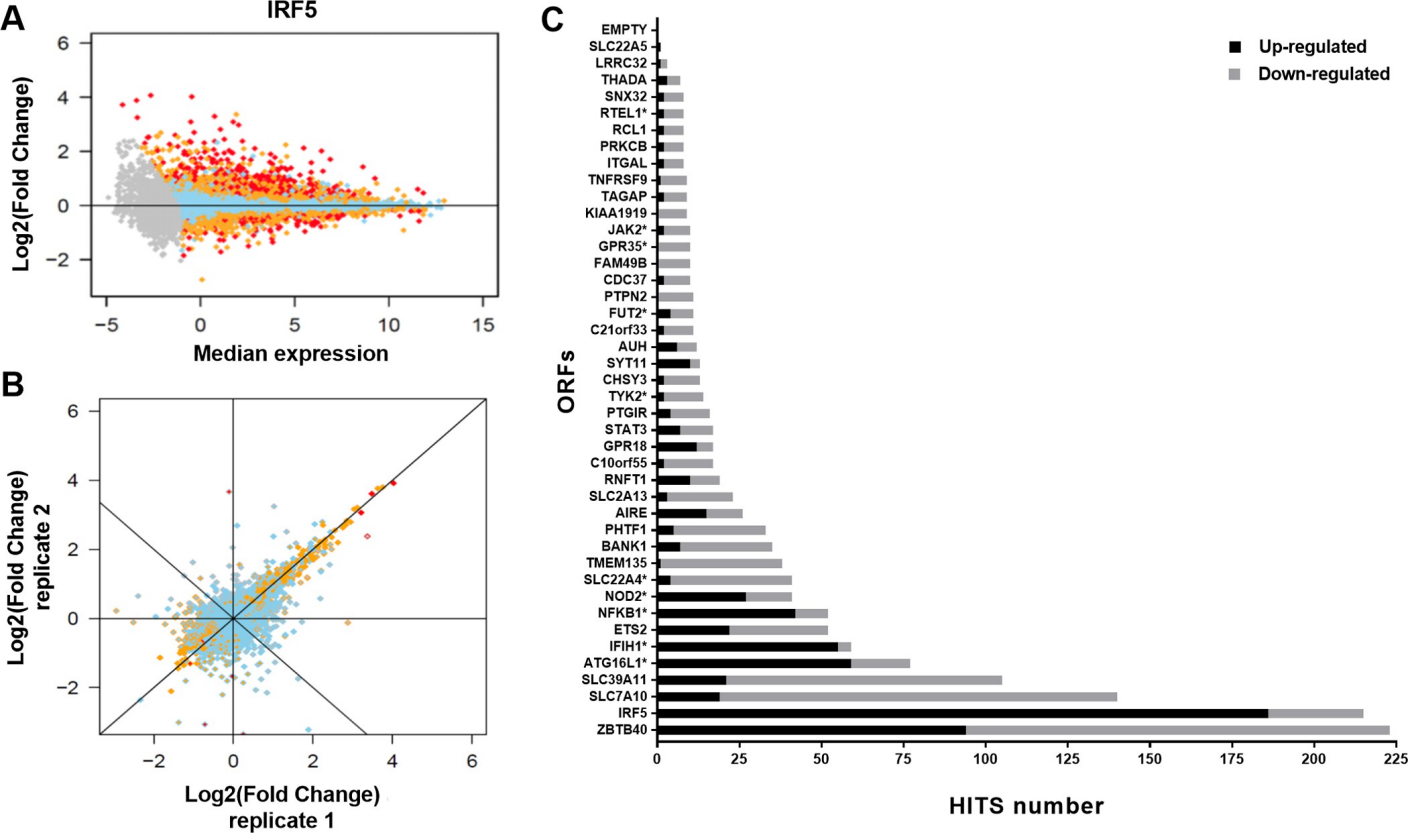
Impact of IBD gene candidate ORFs on the THP-1 transcriptome. **(A)** Selected example illustrating impact observed on the transcriptome of THP-1 cells following the expression of IRF5. Each dot represents a single detectable gene in the THP-1 transcriptome. The x-axis shows the log_2_-transformed median expression across all conditions tested (baseline). The y-axis represents the effect of transduction and expression of a given ORF, as the log_2_-transformed fold-induction compared to baseline. Skyblue dots represent genes with expression value within expected variation (|Z|≤2), orange dots represent genes suggestively outside the range (|Z|>2) and red dots represent genes outside expected range of variation (|Z|>4). Gray dots are genes with expression value below our detection threshold. Additive effect in log2 correspond to multiplicative effect on the original scale. The fold-change equivalent to a given effect log2-effect x is then: FC = 2^x^. As an example, an effect of 1 correspond to a FC = 2. **(B)** Correlation of effect of independent sets of replicated expression of IRF5 on THP-1 transcriptome. The x-axis (inner color of dots) and y-axis (border color of dots) show the effect of two independent set of replicated ORFs on the transcriptome, as the log_2_-transformed fold-induction compared to baseline. Variation between sets of replicates includes effect of independent infection dates, RNA extraction, expression arrays and batches. **(C)** Impact of the transduction and expression of all 42 IBD gene candidate ORFs on the transcriptome of THP-1 cells. ORFs are ordered by their total number of HITS, with the number of up- and down-regulated HITS illustrated by black and gray, respectively **(S2 Table & S1 Appendix**). Starred ORFs are previously reported IBD candidate causal genes.

To better understand the functional impacts of IBD genes in disease susceptibility within the monocyte/macrophage cell compartment, we performed an expression-based screen of genes from known IBD loci. In order to define the genes to include in this transcriptomic screen, we began with the 167 IBD-associated loci that had been identified prior to the initiation of the current study [12] and prioritized 64 genes with endogenous expression profiles consistent with having a role in monocyte/macrophage functions (S1 Fig). Of these, we successfully cloned and stably transduced 45IBD-ORFs into the THP-1 monocytic cell model, each via three independent infections (**S1 Table**). The RNA from these stable cultures was analysed by bulk RNA sequencing. Following quality control, normalisation of this transcriptomic data, merging of samples from different experimental batches and merging of experimental replicates, the induction level (**S2 Table**) and impact on the cell’s transcriptome of 42 of these IBD-ORFs was assessed. To do so, we determined the variance in expression of all detectable genes in the transcriptome across all the samples included in these analyses, and then determined the set of genes that increased or decreased in response to the expression each ORF–these “HITS” were defined as genes where the fold effect computed from the combined replicates was larger than two and the expression was outside the expected range of variation (**Figs 1A & S3A and S3B Table**).

Importantly, there was strong correlation between the replicates of any given ORF, highlighting the robustness of the experimental approach used **(Fig 1B)**. We observed that the different IBD-ORFs showed a wide range of effects on the cell’s transcriptome (*from 1 to 223 HITS*), with IBD-ORFs encoding for known transcription factors (e.g. *ZBTB40*, *IRF5* and *IFIH1*) showing some of the greatest impacts on the transcriptome. On the other hand, ORFs encoding terminal enzymes in a metabolic pathway or proteins whose function likely requires an external stimulus, such as for the known causal genes *FUT2* (11 HITS) or *TYK2* (14 HITS), respectively, only had modest impacts on the transcriptome (**Figs 1C, S3A Table & S1 Appendix**).

### Expression of *IRF5* has a major impact on the THP-1 transcriptome

As a first step to interpreting the results from this ORF-based expression screen, we examined the results obtained for the ORF of interferon regulatory factor 5 (*IRF5*) as it is a gene that encodes a known transcription factor belonging to the interferon regulatory factor family of genes that are highly expressed in human monocytes, macrophages, dendritic cells and B cells [18]. IRF5 plays a central role in inflammation by inducing the production of proinflammatory cytokines and promotes M1 macrophage polarization by directly inducing the transcription of M1 genes [19].

In this screen, the lentiviral transduction of the *IRF5* ORF increased the overall expression of this gene in THP-1 cells by ~3-fold and resulted in the highest number of upregulated HITS (n = 186) **(Fig 1 and S2 Table).** This increased expression of *IRF5* resulted in a ~10-fold or greater increase in the expression of proinflammatory mediators that belong to C-C motif chemokine ligand family (*CCL4*, *CCL4L1*, *CCL8*, and *CCL3*) and of the genes encoding the receptor for the proinflammatory cytokine IL18 (*IL18RAP* and *IL18R1*) **(S3A and S3B Tables**). Notably, *CCL4* is also known to be upregulated and secreted by M1 macrophages. In addition, the increase in *IRF5* expression induced the expression of other M1 macrophages genes, such as the inflammatory cytokine *IL1B* and surface proteins CD83 and *CD40* implicated in antigen presentation and T cell activation, respectively **(S3A and S3B Table**) [19].

A global annotation analysis of the 186 genes that were upregulated following the increased expression of *IRF5* found significant enrichment of multiple terms, most of which are known to be implicated in the function of monocytic and other myeloid cells, including “defense response”, “innate immune response”, “response to cytokine” and “response to virus” (**S4A & S5A Tables**). In addition, the analysis of the proximal promoters of these 186 upregulated genes, by two different tools (gProfiler and PRIMA), revealed significant enrichment for different IRF transcription factor binding sites (TFBS) **(S6 & S7 Tables**), including *IRF5* itself and one of its HITS, *IRF7*. A significant enrichment for *TFBS* of the *STAT1*::*STAT2* heterodimer was also observed. *STAT1* is also a HIT upregulated by *IRF5*. Taken together, these results support that our experimental approach has the capacity to identify ORF-related functions relevant to myeloid biology.

### Multiple IBD genes have shared impacts on the THP-1 transcriptome

We extended the global analysis to the HITS of all the IBD ORFs and identified multiple other ORFs that are enriched for many of the same Gene Ontology terms as IRF5, most notably ATG16L1 and IFIH1, **(S4A and S5A Tables)** consistent with their known roles in anti-microbial responses. This shared effect was also observed in the specific list of genes whose expression was altered in response to these ORFs **(S2 Fig**). While more modest, we also observed that the HITS for IBD ORFs ETS2 and ZBTB40 shared a significant GO annotation (immune system process) **(S3A, S3B, S4A and S5A Tables).** Expressing the ORFs of ETS2 or ZBTB40 in THP-1 cells resulted in up- or down-regulation of a common set of genes **(S3 Fig)** primarily involved in the recruitment and extravasation of inflammatory cells [20–24]. While much more limited in terms of shared impact on the THP-1 transcriptome, the three shared HITs of SLC39A11 and NOD2 **(S4 Fig)** all impact on monocyte/macrophage polarization [25–27].

Finally, the group of *ZBTB40*, *NFKB1*, *SLC39A11* and *PTGIR* ORFs shared many annotation terms such as “inflammatory response” and “cytokine production”, with all four sharing the terms “calprotectin” and “iNOS-S100A8/A9 complex” **(S5A and S5B Table)**. Importantly, calprotectin (CP) is a protein complex whose protein subunits are encoded by the *S100A8* and *S100A9* genes and that is secreted by monocyte/macrophages and neutrophils. CP is known to modulate the inflammatory response by stimulating leukocyte recruitment and inducing cytokine secretion [28]. The shared functional impact of these 4 ORFs was observed in the mutual altered genes in response to these ORFs in THP-1 **(S5 Fig).**

### Multiple IBD susceptibility genes are linked to the calprotectin pathway

While there is widespread use of fecal CP as a biomarker of disease severity and mucosal healing in IBD, a link with susceptibility to IBD has not been previously reported [15]. We therefore wanted to validate the screening results that *ZBTB40*, *NFKB1*, *SLC39A11* and *PTGIR* impact *S100A8* and *S100A9* expression. As can be seen in **Fig 1C**, the *ZBTB40* ORF had a greater impact on the THP-1 transcriptome than *IRF5*, although its effect was nearly evenly split between positive (94 HITS with increased expression levels) and negative (129 decreased) effects. The function of *ZBTB40* has not been fully established, although consistent with the results from our screen, BTB-ZF proteins can act as transcriptional activators or repressors [29]. A global annotation analysis of the 94 HITS that increased following the expression of *ZBTB40* in THP-1 indicated significant enrichment of multiple annotation terms most of them are known to play essential roles in myeloid cells such as: “myeloid leukocyte activation”, “neutrophil activation”, “Toll-like receptor binding” and “myeloid cell activation involved in immune response” **(S4A Table**). Consistent with this, the *S100A8* and *S100A9* genes that encode the two protein subunits that form CP were among the top 10 genes upregulated by *ZBTB40*. We then performed an independent set of three transfections of THP-1 with the ORF of *ZBTB40* and observed a consistent increase in expression of *S100A8* and *S100A9* expression (**S7 Fig**).

*NFKB1* is a transcription factor implicated in many biological processes such as inflammation, immunity, differentiation, cell growth, tumorigenesis and apoptosis. *NFKB1* controls the balance in the activation of pro-inflammatory and anti-inflammatory signaling pathways in the gut [30]. *SLC39A11* encodes a zinc transporter that plays a crucial role in the Zn homeostasis, which is necessary for the innate immune system, especially for maintaining the function of macrophages [31]. Finally, *PTGIR* encodes the receptor for prostacyclin (PGI2). While PGI2 has primarily been studied for the treatment of pulmonary hypertension (PAH), due to its effects on smooth muscle relaxation, more recent studies have revealed anti-inflammatory effects as well [32].

**Fig 2:**
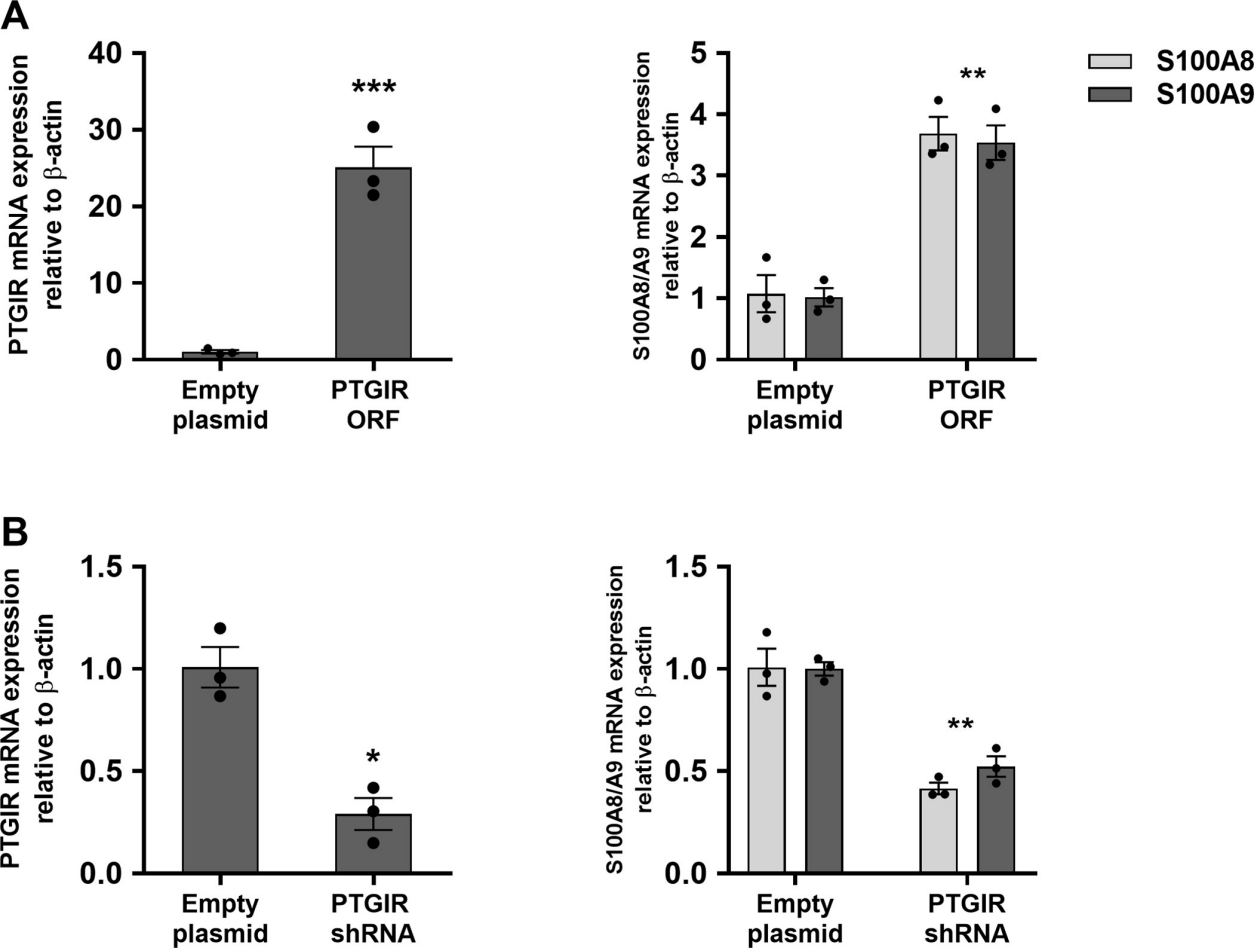
Impact of PTGIR expression and knockdown on S100A8/A9 genes expression in THP-1. **(A)** Relative mRNA expression levels of *PTGIR* and *S100A8/A9* genes in THP-1 cell lines following transduction with lentiviruses containing either an empty plasmid or a plasmid encoding for the *PTGIR* ORF. **(B)** Relative mRNA expression levels of *PTGIR* and *S100A8/A9* genes were evaluated in THP-1 cell lines following transduction with lentiviruses containing either an empty plasmid or a plasmid containing an shRNA targeting *PTGIR* (*PTGIR* shRNA). Each bar is the mean of 3 samples from 3 different infections ±SEM. *P < .05, **P < .01, ***P < .001 (Student’s *t*-test unpaired).

Given the availability of agonists and antagonists for *PTGIR*, we focused our functional studies on this pathway. As a first step, we performed an independent set of three transfections of the THP-1 line with the ORF of *PTGIR*. This resulted in an important induction in the RNA expression levels of both *S100A8* and *S100A9* **(Fig 2A),** as originally observed in the transcriptomic screen. Conversely, knocking down the endogenous expression of *PTGIR* in THP-1 cells by approximately 80% led to a 50% reduction in the RNA expression levels of *S100A8* and *S100A9* (**Fig 2B**). Next, we evaluated the impact of *PTGIR* on CP protein expression. Specifically, we observed significant increases in CP levels in cell lysates (FC = 6.7; *P* = 1.17x10^-3^) and secreted into the culture supernatants (FC = 2.9; *P* = 1.11x10^-3^) in THP-1 following expression of the *PTGIR* ORF (**S8 Fig**). Together this confirms that *PTGIR* levels impact the levels of CP in THP-1 cells, and that modulation of the transcripts for *S100A8/A9* genes are accompanied with modulation of the protein concentration of S100A8/A9 dimer (CP).

### Genetic and pharmacologic modulation of *PTGIR* provides additional support for a link between *PTGIR* and CP pathways in monocytic cells

**Fig 3:**
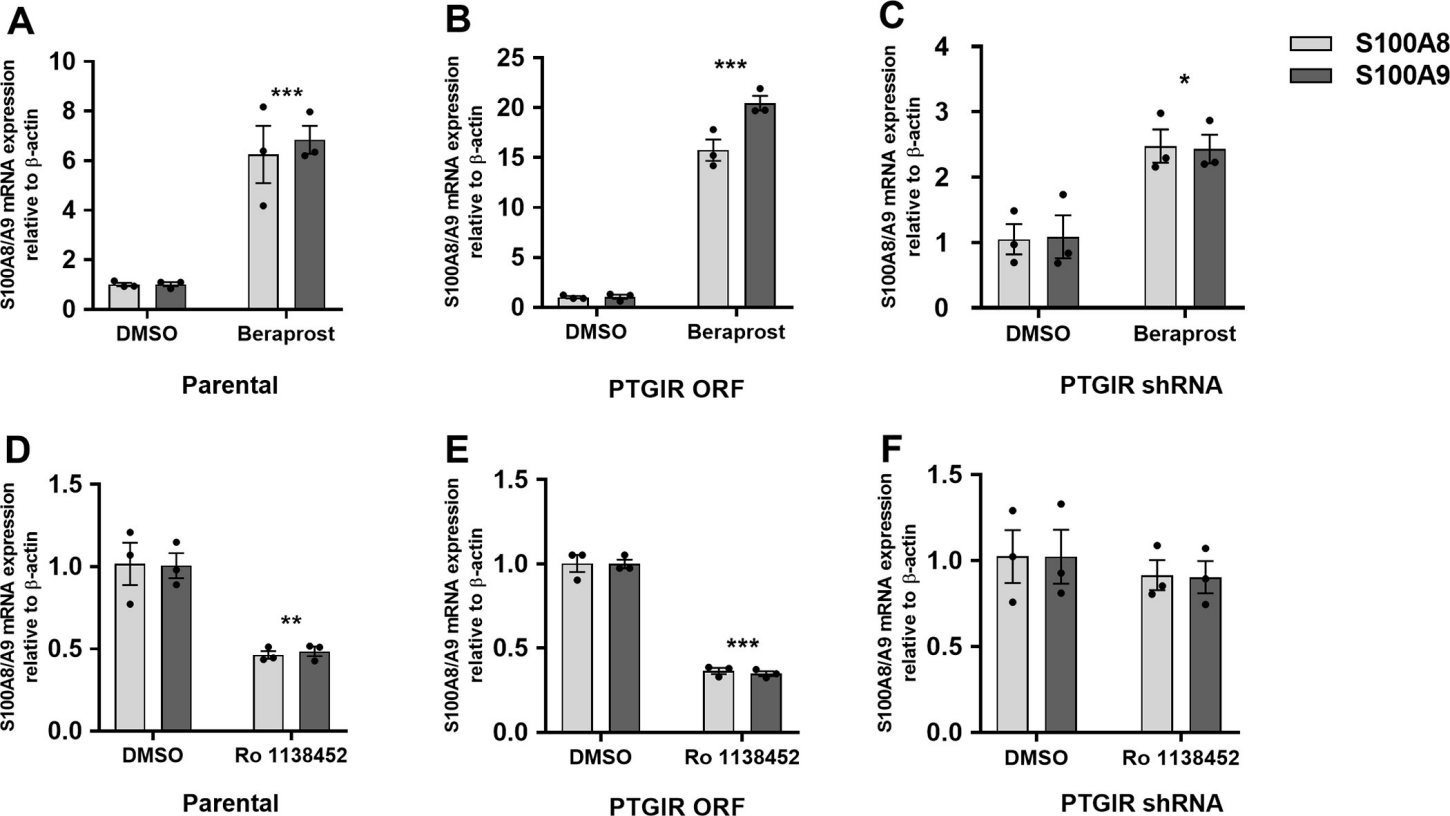
Impact of PTGIR agonist/antagonist on CP genes expression in THP-1: parental, PTGIR expressed or knocked-down. Relative mRNA expression levels of *S100A8/A9* genes were evaluated following the treatment of THP-1 parental cell line **(A)** with 10^−5^ M PTGIR agonist (Beraprost) or **(D)** with 10^−5^ M PTGIR antagonist (Ro 1138452) for 24 h. Relative in mRNA expression levels of S100A8/A9 genes were evaluated in THP-1 cell lines transduced with **(B, E)** PTGIR ORF or (**C, F**) PTGIR shRNA following a treatment with either DMSO and either 10^−5^ M PTGIR agonist (Beraprost) or 10^−5^ M antagonist (Ro 1138452) for 24 h. Each bar is the mean of 3 samples from 3 different infections ±SEM. *P < .05, ***P < .001 (Student’s *t*-test unpaired). A dose and time-course response of *S100A8/A9* RNA expression to PTGIR agonist Beraprost were performed in parental THP-1 cells (**S12 and S13 Figs, respectively**); A dose response of *S100A8/A9* RNA expression to PTGIR antagonist Ro 1138452 was performed in THP-1 cells transduced with the *PTGIR* ORF (**S14 Fig**).

To provide additional support for this novel link between the *PTGIR* pathway and CP production, we studied the effects of well-characterized pharmacologic agents that act as a *PTGIR* agonist (Beraprost) or antagonist (Ro 1138452) on the expression of *S100A8*/*A9*. As can be seen in **Fig 3A**, endogenous levels of *PTGIR* are sufficient for THP-1 cells to respond to the *PTGIR* agonist Beraprost, as measured by the induction of *S100A8* (FC = 6.25, *P* = 9.98x10^-4^) and *S100A9* (FC = 6.85, *P* = 8.24x10^-5^) expression. This response is dramatically increased in THP-1 cells transduced with the *PTGIR* ORF (*S100A8* (FC = 15.75, *P* = 2.18x10^-5^) and *S100A9* (FC = 20.46, *P* = 2.49x10^-4^)) (**Fig 3B**) and is significantly decreased in cells where the expression of the endogenous *PTGIR* has been knocked down (*S100A8* (FC = 2.47, *P* = .02) and S100A9 (FC = 2.43, *P* = .04)) (**Fig 3C**). Furthermore, we found that the increase in *S100A8*/*S100A9* transcript following the stimulation of THP-1 parental cells with Beraprost was accompanied with an increase in secretion of the CP protein in the culture supernatant (FC = 1.67, *P* = .01) (**S8 Fig**). Conversely, the use of the *PTGIR* antagonist Ro 1138452 led to significant decreases of *S100A8* (FC = 0.46, *P* = 5.78x10^-3^) and *S100A9* (FC = 0.48, *P* = 1.67x10^-3^) expression in wildtype THP-1 cells (**Fig 3D**) as well as in THP-1 cells expressing the *PTGIR* ORF (**Fig 3E**). However, no significant change has been observed following treatment with Ro 1138452 in cells where the expression of the endogenous *PTGIR* has been knocked down (**Fig 3F**).

### *PTGIR* signaling leads to transcriptional control of CP genes *S100A8*/*A9* via adenylyl cyclase dependent STAT3 signaling

**Fig 4:**
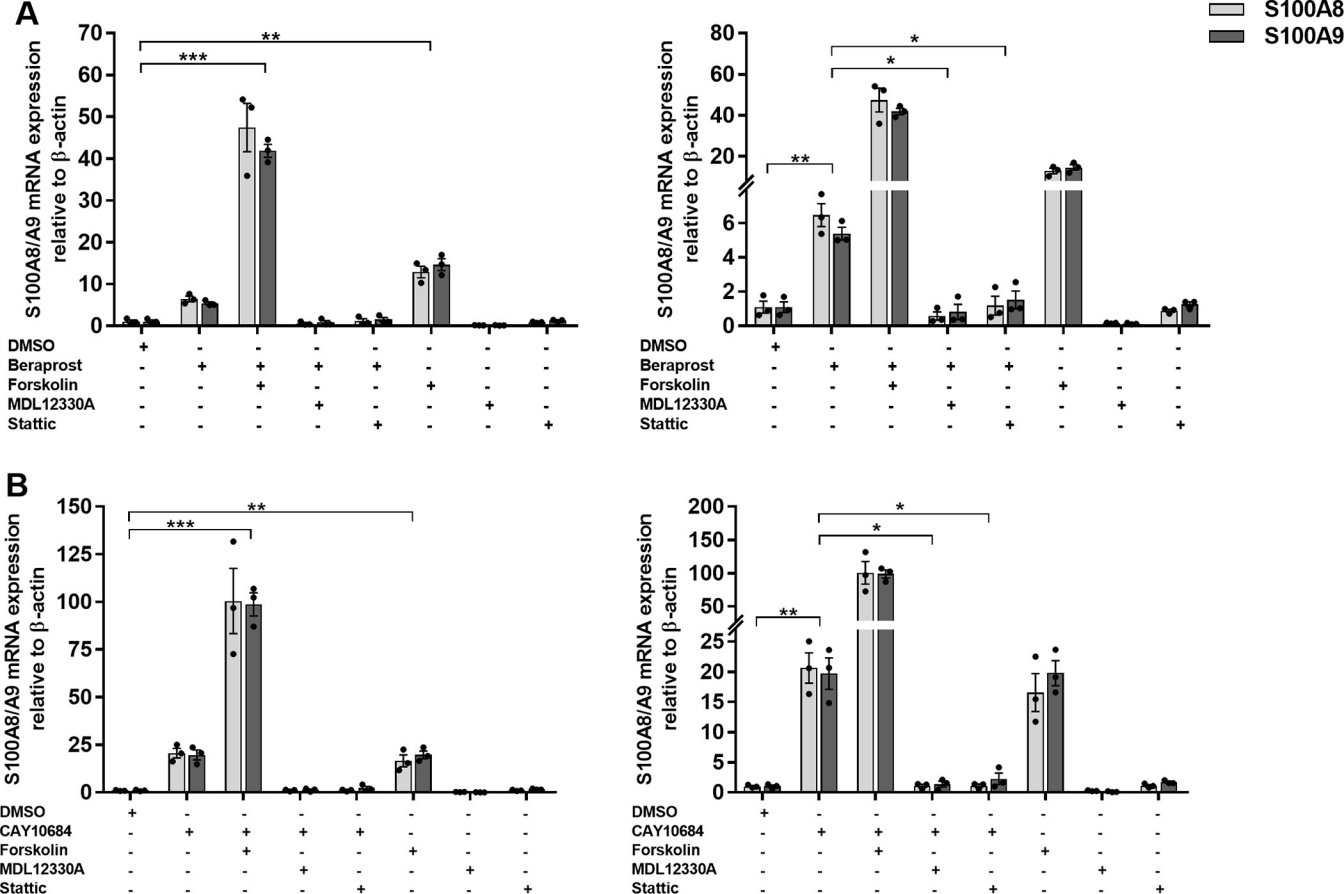
Impact of AC activator/inhibitor and STAT3 inhibitor on the *S100A8/A9* expression induction in parental THP-1. Relative mRNA expression levels of *S100A8/A9* genes were evaluated after treating 5x10^5^ THP-1 cells with either 10μM AC activator (Forskolin), 10μM AC inactivator (MDL12330A) or 2x10^-5^ M STAT3 inhibitor (Stattic) in the presence or absence of **(A)** 1x10^-5^ M PTGIR agonist (Beraprost) or **(B)** 1x10^-5^ M PTGER4 agonist (CAY10684) for 24h. Graphs on the right represents the same data with different y-axis scale. Each bar is the mean of 3 samples from 3 different experiments ±SEM. *P < .05, **P < .01, ***P < .001 (Student’s *t*-test unpaired).

It is known that *PTGIR* is a seven-transmembrane G-protein coupled receptor (GPCR) that is coupled to Gαs and that once this receptor is activated by its ligand, PGI2, the Gαs activates the adenylyl cyclase (AC), which converts the GTP into cAMP [33]. In turn, the cAMP activates PKA, which then stimulates the activity of transcription factors such as CtBP1 [34], SPI [35] and STAT3 [36] by phosphorylation. As *S100A8/A9* appeared to be co-regulated in our dataset and in other studies [37], we evaluated their proximal promoters for the presence of shared TFBS. Specifically, using the publicly available ENCODE chip-seq data, we identified seven different TFs (CTCF, EP300, FOS, POLR2A, REST, SPI1 and STAT3) that had evidence of binding to both the *S100A8/A9* promoters (**S9 Fig**). Of these seven, only STAT3 is known to be activated via the cAMP-PKA pathway and thus constituted our best candidate [38]. Given that transcription control mechanisms can vary from one cell type to another, and that the ENCODE ChIP-seq data for STAT3 was generated using the human epithelial cell MCF10A, we sought to validate this candidate pathway in THP-1 cells. As a first step, we studied the impact of the addition of 10μM Forskolin, a known activator of AC, on the induction of *S100A8/A9* expression in parental THP-1 cells. We found that this activation of AC led to a pronounced increase in expression of both *S100A8* (FC = 15, *P* = 1.41x10^-3^) and *S100A9* (FC = 19, *P* = 9.16x10^-4^), which was potentiated by the addition of the *PTGIR* agonist Beraprost (S100A8, FC = 47, *P* = 2.85x10^-4^; S100A9 FC = 41, *P* = 1.13x10^-5^) (**Fig 4A**). In contrast, we found no induction of *S100A8* or *S100A9* gene expression in cells treated by the AC inhibitor MDL12330A, in the presence of Beraprost (**Fig 4A**). Finally, we tested the role of STAT3 in this signaling pathway by treating the THP-1 parental cells with the STAT3 inhibitor Stattic. Specifically, treatment of the THP-1 cells with 2.10^−5^ M Stattic abrogated the response to the PTGIR agonist Beraprost (**Fig 4A**). These results validate that activation of the prostacyclin pathway leads to an increase in the expression of S100A8/A9 via an AC dependent STAT3 signaling pathway.

### The *PTGER4* pathway also regulates *S100A8/A9* expression

Based on our findings that activation of the PTGIR leads to increased expression of CP, as well as the findings that an increased expression of *ZBTB40* not only led to an induction of *S100A8/A9*, but also of three enzymes involved in PG pathway, *PLA2G1B*, *TBXAS1* and *AKR1C2* **(S3A Table)**, we were interested in investigating the involvement of other members of the PG pathway in the control of expression of *S100A8*/*A9*. In particular, the locus containing the *PTGER4* gene, which encodes the PG E2 receptor (subtype 4), was one of the first to be identified as a risk factor for CD, and then shown to be associated with both CD and UC phenotypes [12,17] and is the predominant locus in genetic studies of IBD in African Americans [26,39]. Importantly, in the same study non-coding variants were found to be associated with increased expression level of *PTGER4* and were proposed as causal alleles, an observation that was later confirmed by others [17,40]. Thus, we decided to study the potential impact of PTGER4 signalling on *S100A8/A9* expression in the human myeloid model THP-1. As can be seen in **Fig 4B**, endogenous levels of *PTGER4* are sufficient for THP-1 cells to respond to the PTGER4-specific agonist CAY10684, as measured by the expression of *S100A8/A9*. Indeed, when these cells were exposed to CAY10684, we observed a very important induction in the expression of *S100A8* (FC = 18.9, *P* = 7.97x10^-5^) and *S100A9* (FC = 20, *P* = 2.16x10^-4^), which was equivalent to the induction of S100A8/A9 observed with the AC activator Forskolin. As was observed for PTGIR, the combination of the PTGER4 agonist and Forskolin led to a synergistic increase in *S100A8* (FC = 100.3, *P* = 3.33x10^-5^) and S100A9 (FC = 98.7, *P* = 1.97x10^-5^). Moreover, the induction of *S100A8/A9* by the PTGER4 agonist CAY10684 was abrogated by the addition of the AC inhibitor MDL12330 (**Fig 4B**). Finally, the induction of S100A8/A9 by CAY10684 was also abrogated by the addition of Stattic, the inhibitor of STAT3 (**Fig 4B**), suggesting a similar signaling pathway as was observed for PTGIR.

### The impact of PG receptors on *S100A8/A9* in response to LPS

**Fig 5:**
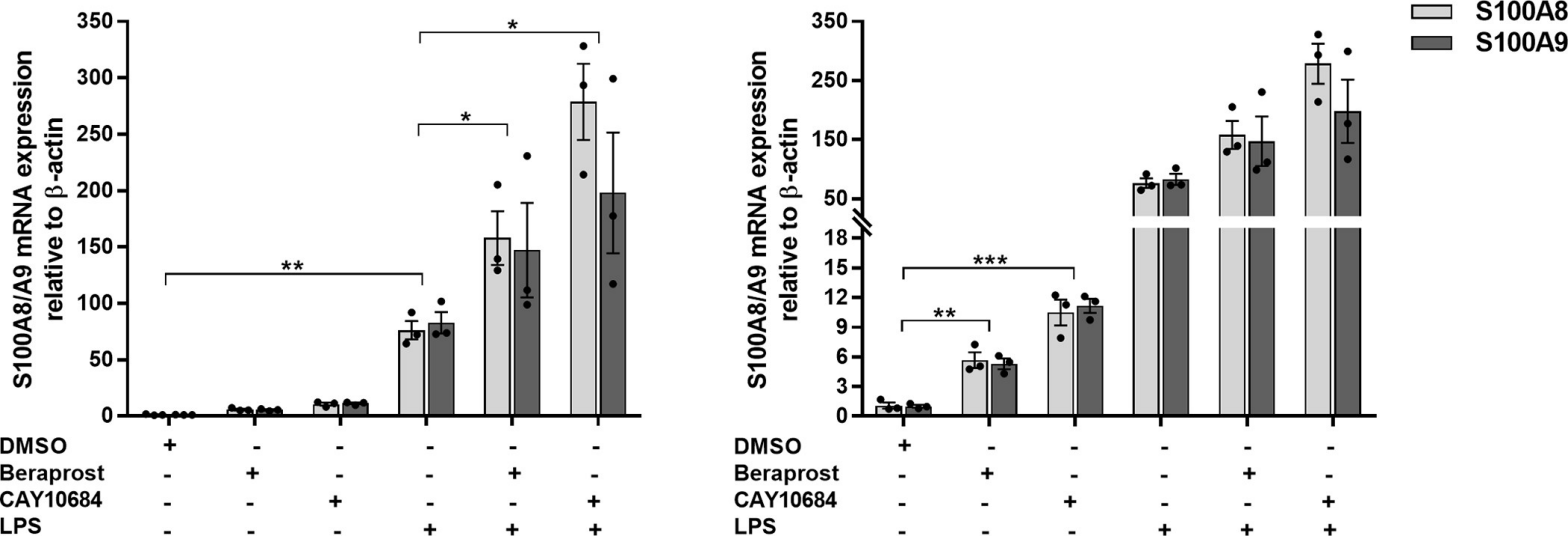
Impact of PG receptor agonists on the expression of *S100A8/A9* in response to LPS. Relative in mRNA expression levels of *S100A8/A9* genes were evaluated after incubating THP-1 for 24 hours with or without 0.2 ug/ml of LPS in the presence or absence of 1x10^-5^ M of Beraprost or CAY10684, the agonists of PTGIR and PTGER4 respectively. Graph on the right represents the same data with different y-axis scale. Each bar is the mean of 3 samples from 3 different experiments ±SEM. *P < .05, **P < .01, ***P < .001 (Student’s *t*-test unpaired).

We also studied the effect of the PG receptor agonists on *S100A8/A9* in THP-1 stimulated by LPS. As can be seen in **Fig 5**, LPS induced the expression of *S100A8/A9*, and also induced the expression of PTGIR **(S10 Fig).** In addition, PTGIR and PTGER4 agonists showed synergistic effect on S100A8/A9 expression in THP-1 when stimulated by LPS. Specifically, *S100A8/A9* was significantly induced when THP-1 cells were treated with the PTGIR agonist in the presence of LPS (S100A8: FC = 2.39, *P* = .047; S100A9: FC = 1.51, *P* = .014) or with the PTGER4 agonist in the presence of LPS (S100A8: FC = 4.44, *P* = .032; S100A9: FC = 2.39, *P* = .044) **(Fig 5)**.

### Validating the role of prostaglandin pathways on the control of CP in human hiPSC-derived monocytes

**Fig 6:**
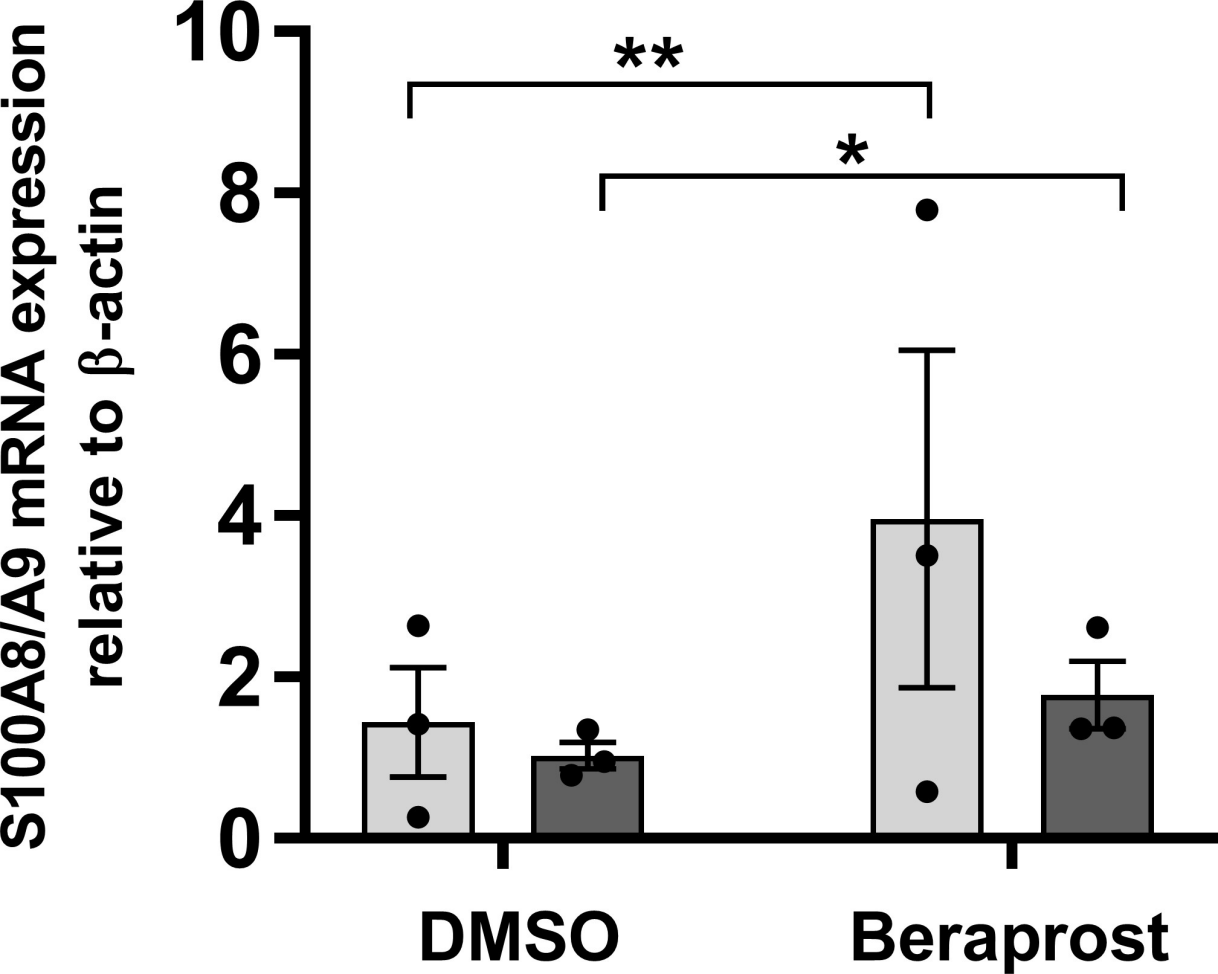
Impact of PTGIR agonist on the expression of CP genes in hiPSC-derived monocyte. **(A)** Relative in mRNA expression levels of *S100A8/A9* genes were evaluated following the treatment of hiPSC CD14+ with 10^−5^ M PTGIR agonist (Beraprost) for 24 hours. Each bar is the mean of 3 samples from 3 different experiments ±SEM. *P < .05, **P < .01 (Student’s *t*-test unpaired).

Although THP-1 are widely used as a model of human monocytes, they derive from a patient with acute monocytic leukemia, thus to validate the PG-CP connection in non-leukemic cells, we studied the impact of the PTGIR agonist on the expression of the CP genes in hiPSC-derived monocytes. Specifically, we found that the treatment of hiPSC monocytes with the PTGIR agonist Beraprost resulted in a significant induction of *S100A8* (FC = 2.34, *P* = 8.89x10^-3^) and *S100A9* (FC = 1.74, *P* = .026) gene expression, providing additional support for this pathway **(Fig 6)**. In an independent experiment, stimulation of macrophages with LPS induced the expression of PTGIR and PTGER4 (**S11 Fig**).

## Discussion

Large-scale comprehensive collaborative studies have mapped out an important portion of the complex genetic architecture of susceptibility to CD and UC, with over 200 genomic loci being associated with CD, UC or both. These genetic findings have helped to identify key biological pathways involved in disease susceptibility ranging from autophagy to cytokine signaling in immune cells as well as the contributions of epithelial cells to anti-microbial control and intestinal barrier functions [41–45]. Moreover, the relevance of these pathways is underpinned by the fact that many are targets of current therapies such as anti-integrins, anti-IL12/IL23 agents and JAK inhibitors. Given that the causal gene has been identified in only a fraction of the known IBD loci, as well as the importance of monocyte/macrophage lineage in IBD pathophysiology, we performed a functional screen of 42 genes from IBD loci in a human cell line often used as a model system for the study of human monocyte/macrophage functions. The scientific premise for the screen was that by modulating the expression of genes from IBD loci that have endogenous expression within this cell lineage, clues to their biological functions could be, at least in part, derived from their impact on the cells’ transcriptome and thus provide potential functional links to the pathogenesis of IBD.

Our analyses of IRF5, one of the 42 genes studied, illustrate this approach. Specifically, the transduction of the ORF for IRF5 in THP-1 cells led to an important increase in expression of genes associated with the polarization towards proinflammatory macrophages, consistent with its known functions [19]. Previous fine mapping of the IRF5 locus defined a region that contained three potential causal genes (*KCP*, *IRF5* and *TNPO3*) [14], and we propose that our findings support *IRF5* as the causal gene within this locus. Moreover, *IRF5* has been associated with susceptibility to different inflammatory and autoimmune diseases including rheumatoid arthritis, systemic lupus erythematosus, multiple sclerosis, and Sjörgen’s syndrome [46]. This is further supported by animal models where a reduction in *IRF5* expression attenuated colitis in mice, but also led to impaired clearance of intestinal pathogens, supporting a modeltogether where homeostasis requires a fine balance in the immune system’s response to gut flora [47].

**Fig 7:**
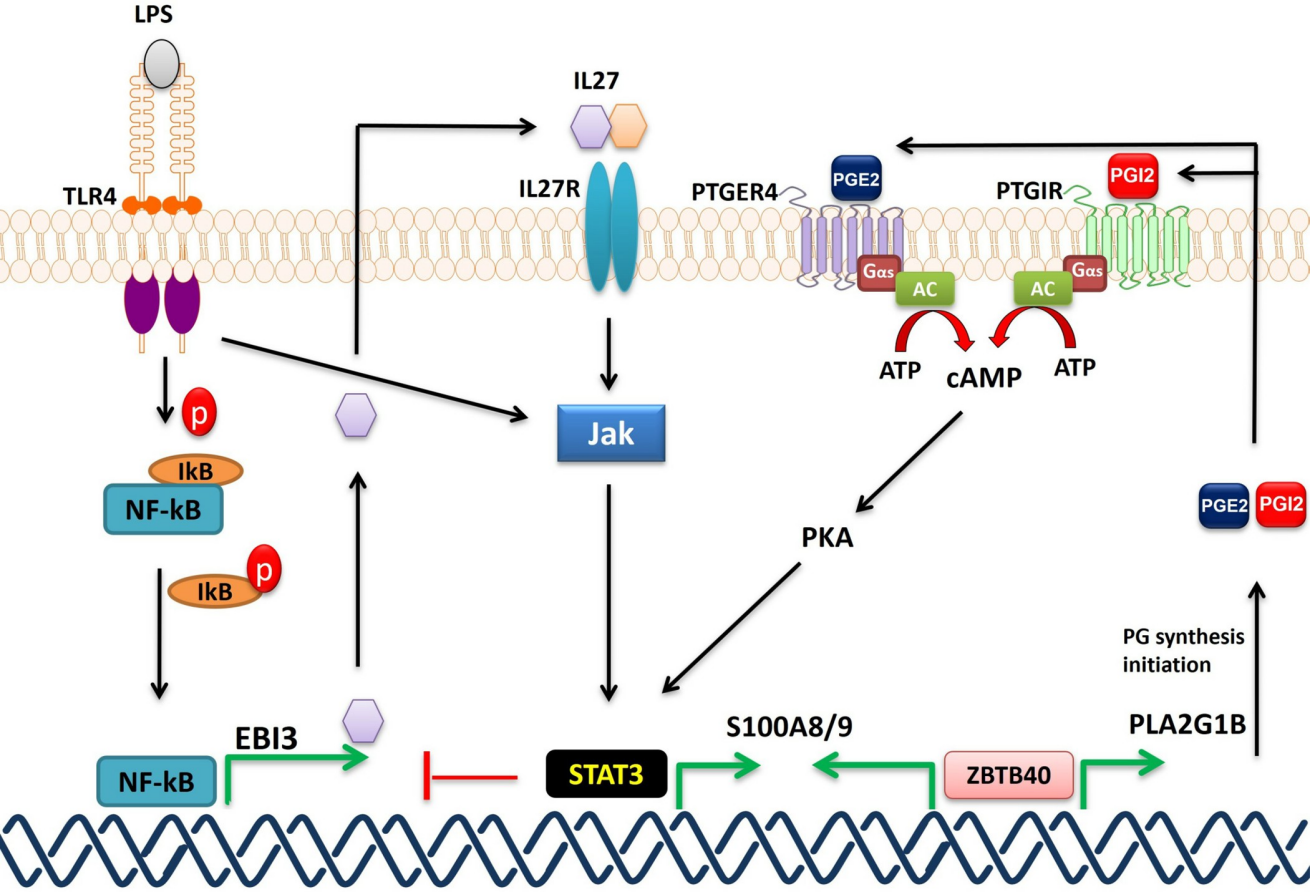
Proposed model of S100A8/9 induction. PTGIR and PTGER4 are activated by their ligands (PGI2 and PGE2 respectively) turning on the AC which converts ATP into cAMP; cAMP activates PKA which in turn triggers STAT3 by phosphorylation. STAT3 binds to the promoters (**S9 Fig**) of S100A8 and S100A9 inducing their expression. TLR4 and when activated by its ligand LPS, activates the JAK2-STAT3 pathway which induces the expression of S100A8/9 and inhibits NF-kB. We have shown the induction of S100A8/9 genes in THP-1 activated by LPS **(Fig 5)**. ZBTB40 could potentially bind directly to the S100A8/9 promoters inducing their expression and/or to the PLA2G1B promoter (***by Encode***) activating its expression. PLA2G1B encodes an enzyme that initiates the PG synthesis including PGI2 and PGE2 which bind PTGIR and PTGER4 respectively, inducing S100A8/S100A9 (see above). Based on RNAseq data, expression of NFKB1 in THP-1 induces the expression of EBI3 (one of the top 5 HITS) (**S3A Table**) most likely via direct binding to the EBI3 promoter by RELB subunit. EBI3 encodes the interleukine-27 subunit beta, which can bind to IL27R and activate JAK/STAT3 pathway [55,56]. In addition, NFKB1 induces the expression of PTGIR (<2 FC, RNAseq data) and by LPS **(S10 Fig).** RELB binds directly to PTGIR promoter (***by Encode***).

The results described herein also revealed a connection between four genes within distinct IBD loci (*ZBTB40*, *SLC39A11*, *NFKB1*, and *PTGIR*) and the expression of the *S100A8/A9* CP genes. CP is a proinflammatory protein and its concentration in fecal and serum samples was shown to reflect IBD severity, although its utility as a biomarker still requires additional technical and clinical validation [48]. Given this, we chose to further validate this link to IBD susceptibility genes. Specifically, we have demonstrated that the upregulation of *PTGIR* expression in THP-1 induces the expression of the *S100A8/A9* encoding for the two CP subunits and stimulates the secretion of CP protein. In addition, we showed that activating parental THP-1 cells with a PTGIR agonist increased *S100A8/A9* expression as well as the intracellular CP protein level, while a PTGIR antagonist resulted in reduced *S100A8/A9* RNA expression levels. Thus, PTGIR signaling has an important impact on CP RNA and protein expression, as well as on its secretion into the extracellular milieu. Moreover, we have validated the pathway by which the PTGIR induces the CP genes in our model. We have shown that the activation of the PGI2 receptor by its agonist induces the expression of *S100A8/A9* genes by activating AC, which is known to trigger cAMP-PKA pathway [49]. PKA is known to activate STAT3 via phosphorylation [36]. Activated STAT3 can then bind to the regulatory elements of *S100A8/A9* genes [50], inducing their expression. Although not part of the screen described herein, we extended our functional analyses to the PTGER4 receptor as it is part of the prostaglandin signaling pathway, albeit having a distinct ligand to PTGIR, and because it is a known causal IBD gene. Indeed, our results also provided support for the PGE2-PTGER4 pathway in the control of CP synthesis in monocyte/macrophages, which is consistent with studies in prostate epithelial cells **[51]. We further demonstrated that** PGE2-PTGER4 signaling is also mediated by cAMP to PKA to STAT3 signaling, elucidating a common signaling pathway for two IBD genes and CP production (**Fig 7**). Although the THP-1 cell line is a widely used model for studies of macrophage differentiation, activation, and innate immune functions, we sought to validate our findings in hiPSC-derived monocytes as they are a renewable source of non-cancerous cells with normal ploidy. Again, we observed that stimulation of these with a PTGIR agonist resulted in an increased production of CP. Importantly, the production of CP triggered by the PTGIR or PTGER4 ligands, that was observed in THP-1 cells, was potentiated by the addition of LPS **(Fig 5).** This was likely due to the increased expression of both receptors in response to LPS as we observed in THP-1 and hiPSC-derived macrophages **(S10 and S11 Fig).** It should be noted that whereas PTGER4 is strongly expressed in monocyte/macrophages and in neutrophils from peripheral blood of healthy individuals, PTGIR expression is high in the former and lacking in the latter, suggesting that the pathway described herein is more specific to the monocyte/macrophages lineage [52].

In terms of genetics, all five GWAS loci are associated with UC (*PTGIR*, *NFKB1*, *SLC39A11*) or with both UC and CD (*ZBTB40*, *PTGER4*) (**S8 Table**). Of these, only the PTGER4 has been fine mapped, with three independent signals that supported *PTGER4* as being the causal gene [14], consistent with an earlier functional study demonstrating that CD-associated variants regulate the expression of *PTGER4* [17]. For the *PTGIR* and *NFKB1* loci, the index SNP is in the 3’UTR and intron 11 of these specific genes, respectively, and are the only genes with their LD regions when defined as the positions of the furthest up-and-downstream variants with an r2 of at least 0.8 to the index SNP (**S8 Table**). For the *ZBTB40* and *SLC39A11* loci, the LD region does not contain any gene. When relaxing the LD region definition to include variants with an r2 of at least 0.5 to the index SNP, the *ZBTB40* locus contains three genes (*ZBTB40*, *WNT4*, *EPH8*), with functional data (published and herein) being consistent with *ZBTB40* being the most likely candidate causal gene in the region. Finally, with this relaxed LD definition, *SLC39A11* is the only protein-coding gene in its locus (**S8 Table**). It should be further noted that *ZBTB40* and *SLC39A11* are the closest genes to their respective index SNPs. This is very relevant given that recent studies have shown that proximity to the index SNP has the best recall when predicting the causal gene within a GWAS locus, with precision and recall being the greatest within 1kb of the index SNP [53,54], which is the situation for *SLC39A11*. While this is not definitive genetic proof of causality, these observations build a case for their causality, especially given their common link to calprotectin expression identified in the current study.

While we did not elucidate how the other IBD genes that were found in our screen to modify CP expression in THP-1 cells, our results do suggest potential mechanisms by which they do so. First, we found that increasing the expression of *NFKB1* led to an upregulation of *PTGIR* expression although just shy of a two-fold threshold (FC = 1.995). *NFKB1*, which encodes a subunit of the well characterized and ubiquitous transcription factor NF-kappa-B, may also act indirectly via transcriptional control of genes such as *TNF* or *EBI3* as observed in the current screen, which would in turn stimulate the JAK2-STAT3 pathway [55,56] leading to increased expression of *S100A8/A9* (**Fig 7**). In addition, the increased expression of *ZBTB40* induced the expression of three genes that encode key enzymes within the prostaglandin pathway (PLA2G1B, *TBXAS1* and *AKR1C2*), thus likely impacting on this pathway. Given that *ZBTB40* is a transcription factor/regulator, it likely functions by binding to some or all the promoter regions of *S100A8*, *S100A9*, *PLA2G1B*, *AKR1C2* and *TBXAS1*. The mechanism by which SLC39A11 impacts on the expression of *S100A8/A9* is more speculative as it encodes a relatively uncharacterized metal ion transporter that is believed to transport zinc ions; thus its role may be linked to its control of zinc which is required to stabilize ZBTB40 and other zinc finger proteins [57]. Interestingly the *SLC39A11* IBD gene led to an increase in expression of the PG E2 receptor subtype 2 (PTGER2).

Finally, together these results support a potential role for the prostacyclin/prostaglandin biogenesis and signaling pathways in IBD susceptibility and pathogenesis. At the present time, there is little known about the role of prostacyclin in IBD although it has been reported that it, as well as PGE2, can regulate lymphatic and vascular functions in the intestine [58]. It has also been demonstrated that PGE2 stimulation of PTGER4 in macrophages leads to the secretion of chemokine (C-X-C motif) ligand 1 (CXCL1), which in turn drives epithelial cell differentiation and proliferation from regenerating crypts, favouring mucosal healing [59]. It should also be noted that CXCL1 is a chemoattractant for neutrophils and its serum level is elevated in CD patients [60,61]. Interestingly, mesenchymal stromal cells (MSCs) may be a potential source of intestinal PGE2, promoting macrophages to adopt an anti-inflammatory activation state [62]. It has also been recently proposed that there is regulatory interaction between IL-10 and PGE2, that when this balance is perturbed, there is aberrant macrophage activation that contributes to IBD pathogenesis [63]. Importantly, calprotectin has been shown to mediate a variety of biological functions that are key to homeostatic antimicrobial defenses but that in the chronic disease state it may exacerbate inflammation in patients with IBD [64].

## Conclusion

In conclusion, the current study provides evidence that five genes (*PTGIR*, *PTGER4*, *ZBTB40*, *NFKB1* and *SLC39A11*) associated with IBD susceptibility are also involved in, directly or indirectly, the control of the expression of calprotectin genes *S100A8* and *S100A9*. This work also supports that prostacyclin/prostaglandin biogenesis and signaling pathways potentially have an important role in IBD pathogenesis.

## Methods

### Ethics statement

The conversion of Human lymphoblastoid cell lines (LCL) collected by the NIDDK Inflammatory Bowel Disease (IBD) Genetics Consortium into hiPSC lines and the use of hiPSC-derived macrophage models in this study were approved by the Montreal Heart Institute Ethics Review Board.

### Selection, cloning and lentiviral transfer of IBD candidate genes from GWAS regions

To identify the IBD gene candidates to test in our transcriptome-based screen for IBD gene functions, we focused on the 163 IBD-associated loci identified by the International IBD Genetics Consortium. First, all genes (297 genes) found within the LD region around each index SNP—as defined by all SNPs having an r2≥0.8 with the index SNP using the SNAP online tool (using the pilot 1000 Genomes dataset in genome build GRCh37/hg19)—were selected. Second, for regions where no genes were identified within the LD region, we selected the closest gene on either side of the index SNP. For IBD-associated regions where the causal gene was already known, only the causal gene was considered. From the combined list of genes identified in this manner, we removed the major histocompatibility complex (MHC) region and HOX gene clusters, as well as genes encoding known secreted proteins (cytokines, chemokines, etc.) and receptors (identified through bioinformatic analysis), as these classes of proteins are unlikely to yield any information in an ORF-expression based screen without their required ligand or receptor. Finally, we removed from this list any gene that did not have detectable RNA expression (minimal log2 transformed expression value of three) in our Agilent expression profiling dataset of human myeloid cells (neutrophil, monocytes, macrophages and macrophages activated by LPS) or cell lines (THP-1) **(S1 Methods)**. In total, 64 were selected from these loci **(S1 Table & S1 Methods).** We cloned the open reading frames (ORFs) for these genes into a modified GATEWAY compatible polycistronic lentiviral expression vector, pLVX-EF1a-IRES-PURO/eGFP, for expression in the THP-1 monocyte cell line. To generate stable cell lines expressing the different candidate ORFs, THP-1 cells were transduced at least in triplicate via spinoculation. Transduction of the whole set of IBD gene candidate ORFs was performed in three batches of about 15 ORFs each, as well as an empty vector control. THP-1 cells were then grown for an average of 7 days (with a range of 4–16 days) to select successfully transduced cells and reach 1x10^6^ cells/ml (3–5 x10^6^ cells in total) before RNA extraction. This was achieved for 45 ORFs1 **(S1 Appendix)**. RNA was then extracted from these samples and sequenced (see below). Following QC and data normalization, 42 ORFs in total were included in the analyses in this study: one failed in RNA sequencing and two ORFs (MARS2 and HHEX) had very large effects on THP-1, which made them hard to analyse in the pipeline and to interpret.

### Preparation of the total RNA library and sequencing

Total RNA was extracted from stably transduced THP-1 cultures using the RNeasy Plus Mini kit (Qiagen) according to manufacturer’s protocol. The RNA samples were quantified, and quality controlled using an Agilent RNA 6000 Nano kit (Agilent) on an Agilent Bioanalyzer 2100 system. Samples with RNA Integrity Number (RIN) below 8 were discarded. RNA sequencing was performed at the McGill University/Genome-Québec Innovation Center. Briefly, RNA samples were transformed into barcoded DNA libraries using TruSeq Stranded mRNA library preparation kits (Illumina), which were then paired-end sequenced, generating 2x100bp reads, using an Illumina HiSeq2000 sequencer. Raw FASTQ sequences were downloaded from the platform’s server for local processing and analysis **(S1 Methods).** After quality control and normalization of the RNAseq data, 42 ORFs in total have been selected to be analysed in this study **(S1 Fig).**

### Bioinformatic annotation of HITS identified in the screen

The set of genes that increased or decreased in response to the increased expression each ORF–named “HITS”—were defined as genes where the fold effect computed from the combined replicates was larger than two, and the expression was outside the expected range of variation based on the entire dataset. In order to find biological categories enriched in HITS identified in the screen (up-regulated, down-regulated, and combined lists), we performed enrichment analyses using the g:GOSt functional profiling tool from the online g:Profiler service [65] these analyses included gene and phenotype ontologies, biological pathways, protein database and regulatory motifs in DNA. In addition, different cis-regulatory motif analyses of the proximal promoters of these HITS have been done using PRIMA method [66] as implemented in the EXPANDER software (v8.0) **(S1 Methods)**.

### Validation of *PTGIR* (expression/knockdown) effect on THP-1

For the validation of the effect of *PTGIR* on THP-1, an independent set of stable cell lines expressing the *PTGIR* ORF were generated via three independent transductions with lentivirus containing the *PTGIR*-ORF or the empty lentiviral plasmid. In addition, three cell lines where the endogenous expression of *PTGIR* was knocked down via lentiviral *PTGIR*-targeting shRNA transfer were generated and for the control the THP-1 cells have been transduced in triplicate with lentivirus containing empty plasmide. The RNA expression levels of *PTGIR*, *S100A8* and *S100A9* genes were quantified by qPCR. **(S1 Methods)**.

### Pharmacologic assessment of *PTGIR* and PTGER4 signaling pathways

THP-1 cells were centrifuged and resuspended in fresh complete media in a 24-well plate at a concentration of 10^6^ cells/mL and grown for 24 hours. The cells were then treated for 24h with different combinations of PTGIR agonist (Beraprost sodium; Sigma), PTGIR antagonist (Ro 1138452 hydrochloride; Tocris), PTGER4 agonist (CAY10684; Cayman), adenylyl cyclase activator (Forskolin; Sigma), adenylyl cyclase inhibitor (MDL12330A; Cayman), STAT3 inhibitor (Stattic; Cayman), or LPS (Sigma-Aldrich).

### Generation, characterization and testing of hiPSC-derived monocytes and macrophages

Human induced pluripotent stem cells (hiPSC) have been generated by reprogramming lymphoblastoid cell lines (LCL) and then differentiated into monocytes **(S1 Methods)**. These monocyte cultures were immunophenotyped by flow cytometry. An enriched population of CD14+ cells was obtained via MACS purification **(S6 Fig).** Independent hiPSC-derived monocytes obtained from different individuals were centrifuged and resuspended in fresh complete media in a 24-well plate at an average of 250,000 cells/mL and then treated for 24h with either PTGIR agonist or carrier. Levels of *S100A8/A9* were then evaluated by qPCR. In an independent experiment, harvested monocytes were also further cultured and differentiated into M0 macrophages by adding M-CSF for 7 days, and then polarized to M1 macrophages with IFNγ. M1 macrophages were then cultured for four hours +/- LPS (100ng/ml) and the levels of PTGIR and PTGER4 were measured.

## Supporting information

S1 Table Selection of genes to include in expression screen. (XLSX)

S2 Table Induction level of the ORFs and the number of the regulated HITS. (XLSX)

S3 Table **S3A Table**: Total HITS Up/Down regulated by each ORF in THP-1 cells. **S3B Table**: Heatmap of HITS of all ORFs in THP-1 cells.
(XLSX)

S4 Table **S4A Table**: gProfiler enrichment analyses of all the HITS identified in the screen. **S4B Table**: gProfiler enrichment analyses of the upregulated HITS identified in the screen. **S4C Table**: gProfiler enrichment analyses of the downregulated HITS identified in the screen.
(XLSX)

S5 Table **S5A Table**: Heatmap of gProfiler enrichment of all the HITS identified in the screen. **S5B Table**: Heatmap of gProfiler enrichment of the upregulated HITS identified in the screen. **S5C Table:** Heatmap of gProfiler enrichment of the downregulated HITS identified in the screen.
(XLSX)

S6 Table Transcription factor binding site analysis of HITS upregulated by different IBD-ORFs by gProfiler. (XLSX)

S7 Table Transcription factor binding site analysis of HITS upregulated by different IBD-ORFs by PRIMA-EXPANDER. (XLSX)

S8 Table Genetic data of the 5 loci of PTGIR, ZBTB40, NFKB1, SLC39A11 and PTGER4. (XLSX)

S9 Table Forward and reverse primer sequences 5´-3´ used in the study. (XLSX)

S10 Table Numeric data for the graphs in the main and the supporting figures. (XLSX)

S1 Fig Flow chart of the expression-based functional screen, with validation steps. (TIF)

S2 Fig Shared impact on transcriptome for the ORFs ATG16L1, IFIH1 and IRF5. (TIF)

S3 Fig Shared impact on transcriptome for the ORFs ETS2 and ZBTB40. (TIF)

S4 Fig Shared impact on transcriptome for the ORFs SLC39A11 and NOD2. (TIF)

S5 Fig Shared impact on transcriptome for the ORFs PTGIR, NFKB1, SLC39A11 and ZBTB40. (TIF)

S6 Fig Generation of a hiPSC-derived monocytic model. (TIF)

S7 Fig Impact of ZBTB40 expression on S100A8/9 genes expression in THP1. (TIF)

S8 Fig Impact of PTGIR expression on the CP (S100A8/9 dimer) protein levels in THP1. (TIF)

S9 Fig Identification of TFBS in the promoter regions of S100A8 and S100A9. (TIF)

S10 Fig Impact of LPS on PTGIR expression in THP-1. (TIF)

S11 Fig Gene expression of PTGIR and PTGER4 in hiPSC monocyte/macrophages derived. (TIF)

S12 Fig The impact of different concentrations of PTGIR agonist on CP genes. (TIF)

S13 Fig The impact of PTGIR agonist on CP genes at different time. (TIF)

S14 Fig The impact of different concentrations of PTGIR antagonist on CP genes expression in THP1. (TIF)

S1 Appendix Impact of the expression of all 42 IBD gene candidate ORFs on the transcriptome of THP-1 cells. Graphs illustrating the impact observed on the transcriptome of THP-1 cells following the expression of each ORF (42 ORFS). In the first four (ZBTB40, SLC39A11, NFKB1, PTGIR), the S100A8 and S100A9 genes are labeled. For the description of graphs see *Methods* *section*.
(PDF)

S1 Methods Supplementary Methods. (DOCX)
