# Supplementary figures and images for: Prostaglandins and calprotectin are genetically and functionally linked to the Inflammatory Bowel Diseases
Source: PLoS Genet. 2022 Sep 26;18(9):e1010189. doi: 10.1371/journal.pgen.1010189 (PMC9536535; doi:10.1371/journal.pgen.1010189)

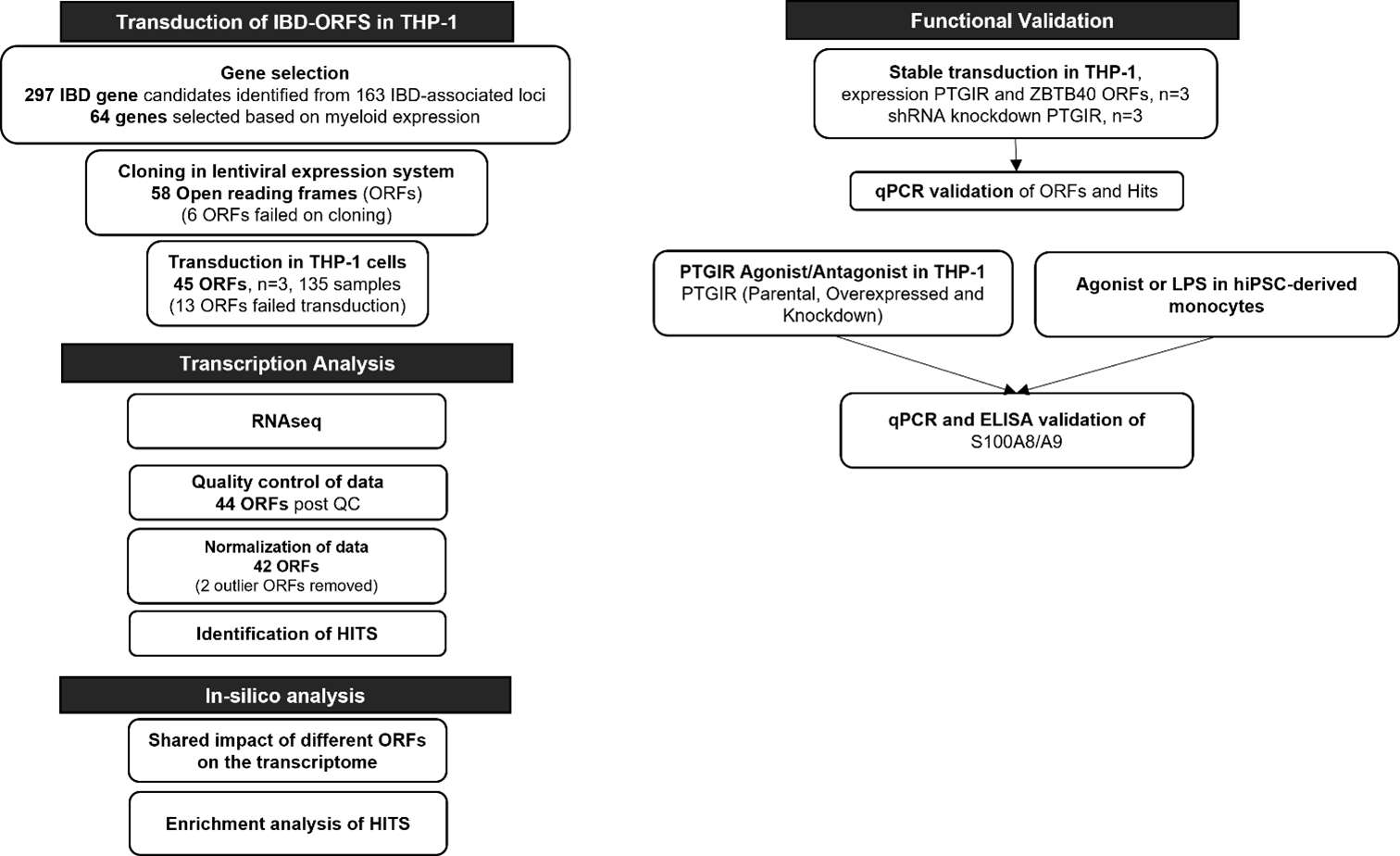

Supplement: S1 Fig — (TIF) [file pgen.1010189.s011.tif]

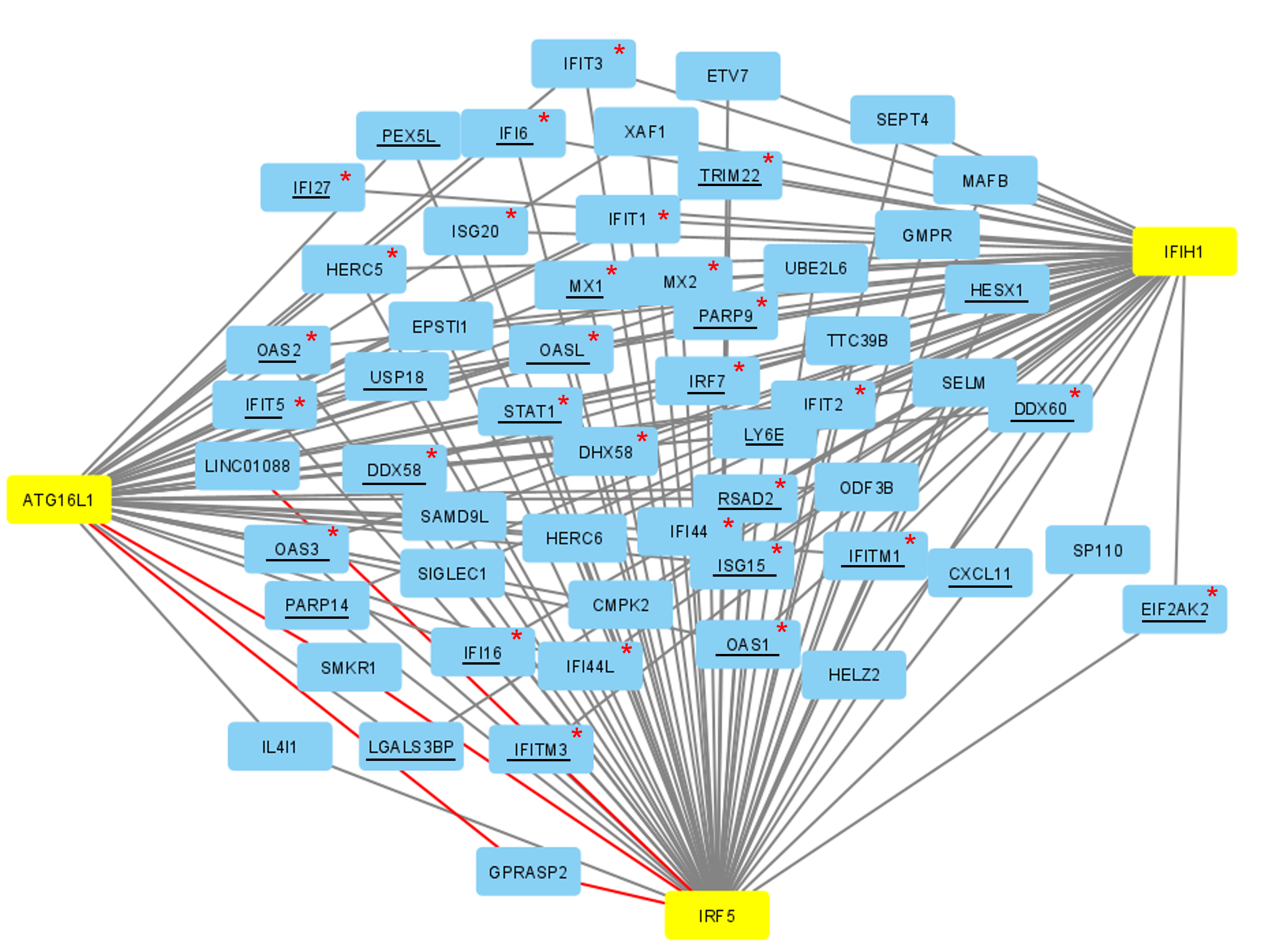

Supplement: S2 Fig — (TIF) [file pgen.1010189.s012.tif]

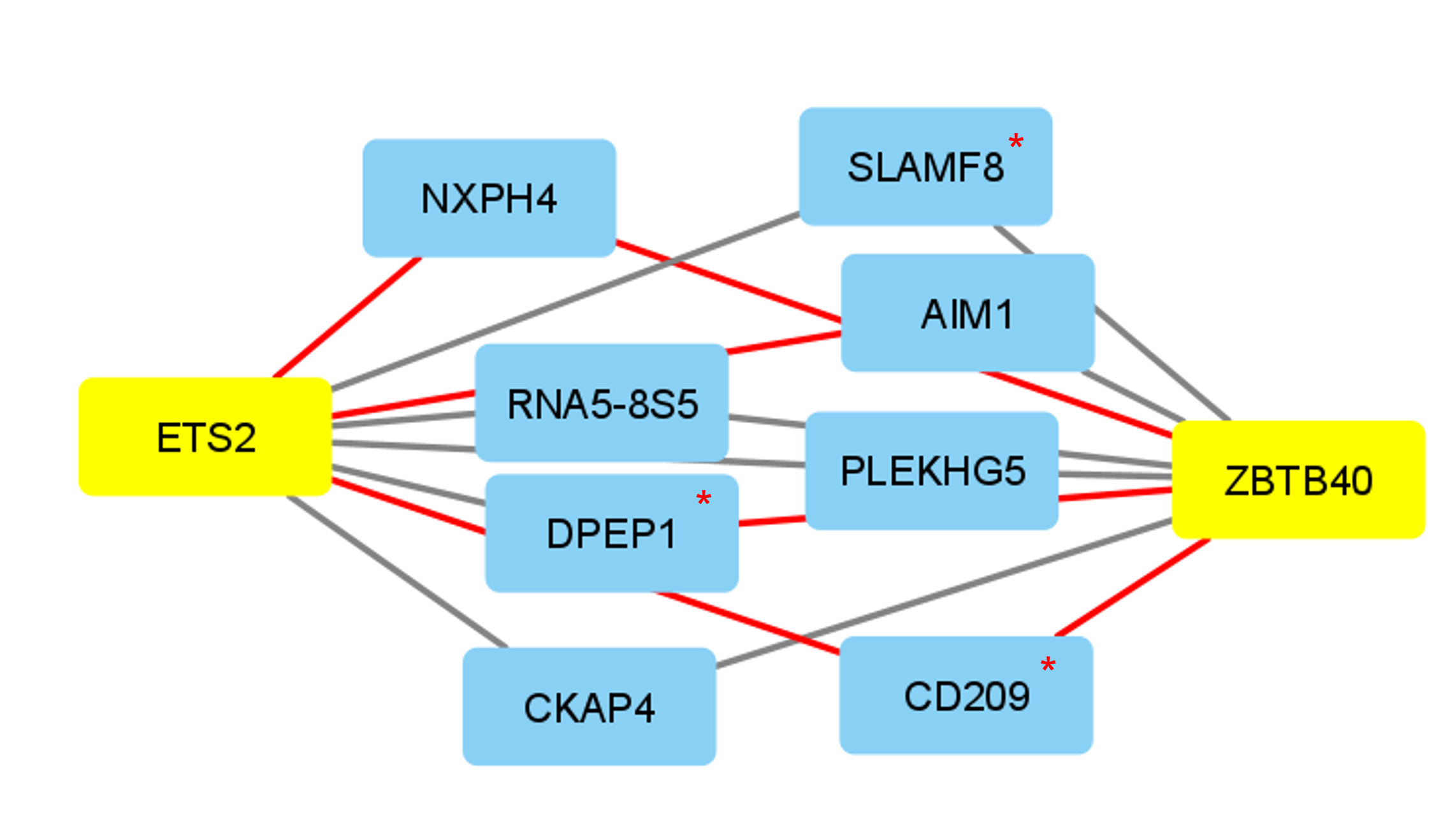

Supplement: S3 Fig — (TIF) [file pgen.1010189.s013.tif]

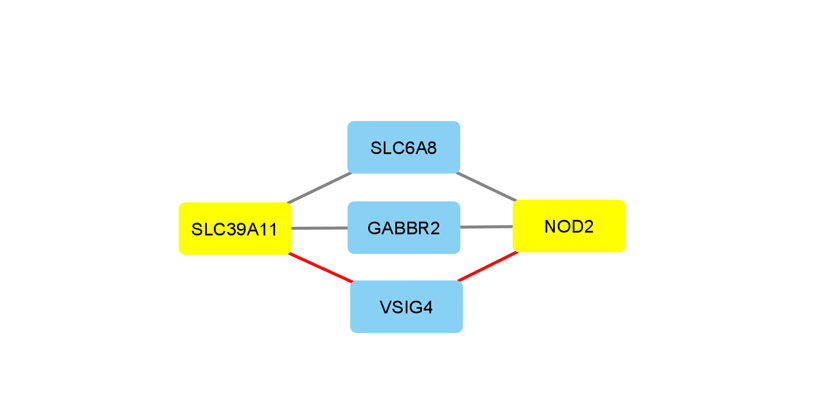

Supplement: S4 Fig — (TIF) [file pgen.1010189.s014.tif]

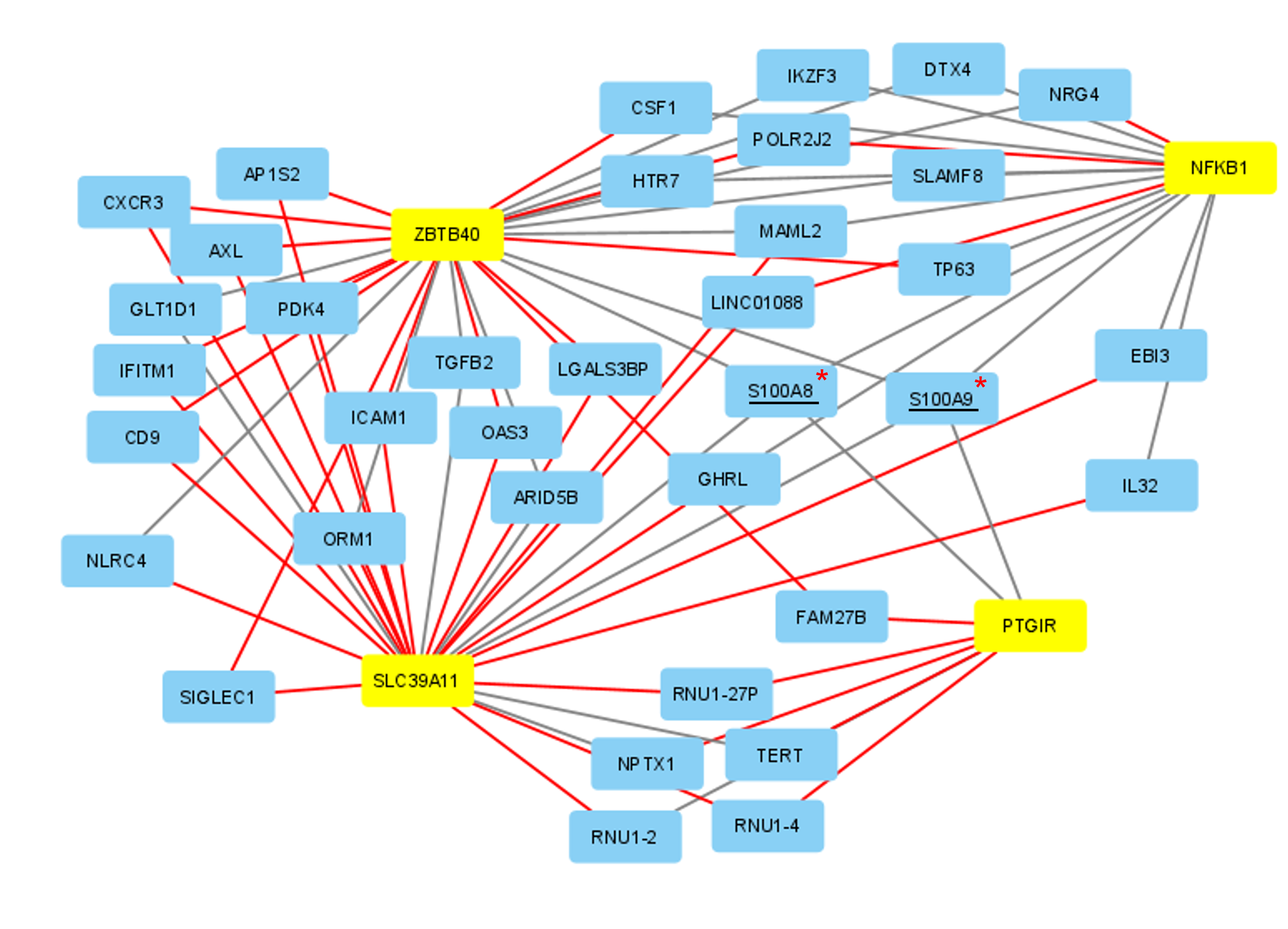

Supplement: S5 Fig — (TIF) [file pgen.1010189.s015.tif]

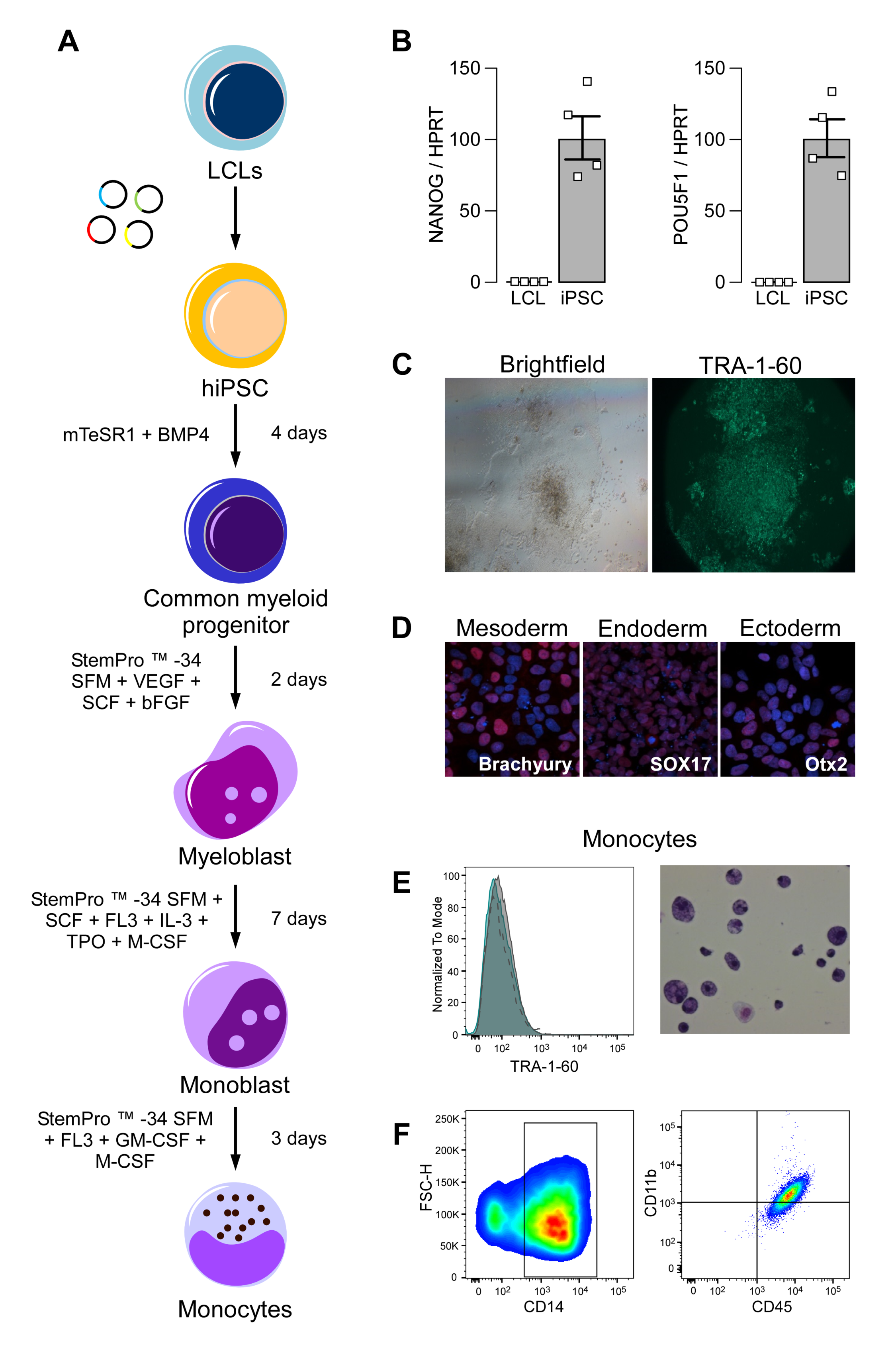

Supplement: S6 Fig — (TIF) [file pgen.1010189.s016.tif]

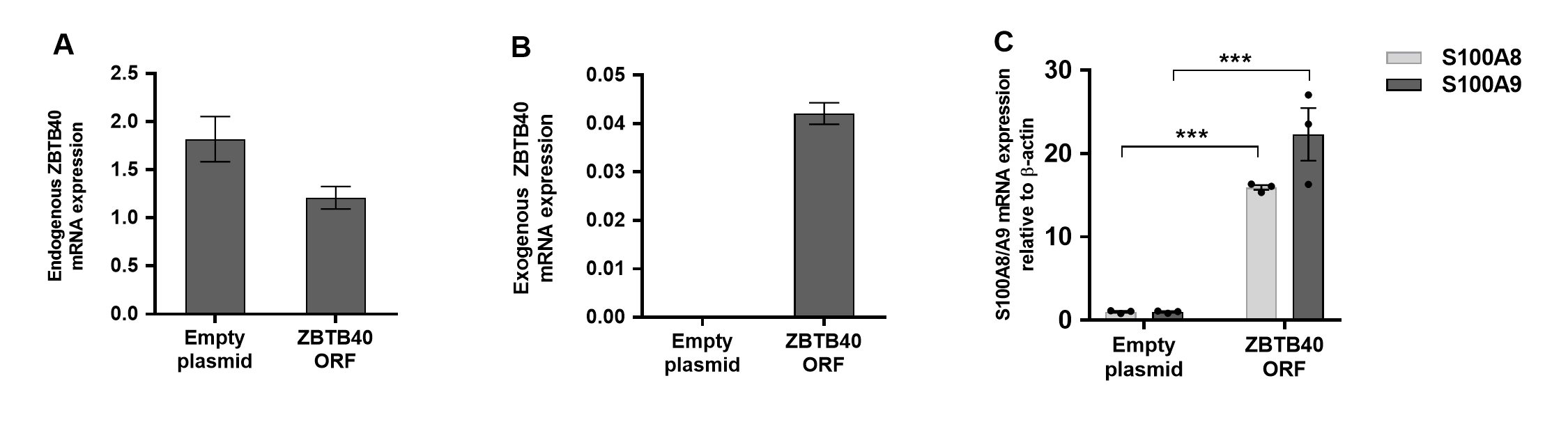

Supplement: S7 Fig — (TIF) [file pgen.1010189.s017.tif]

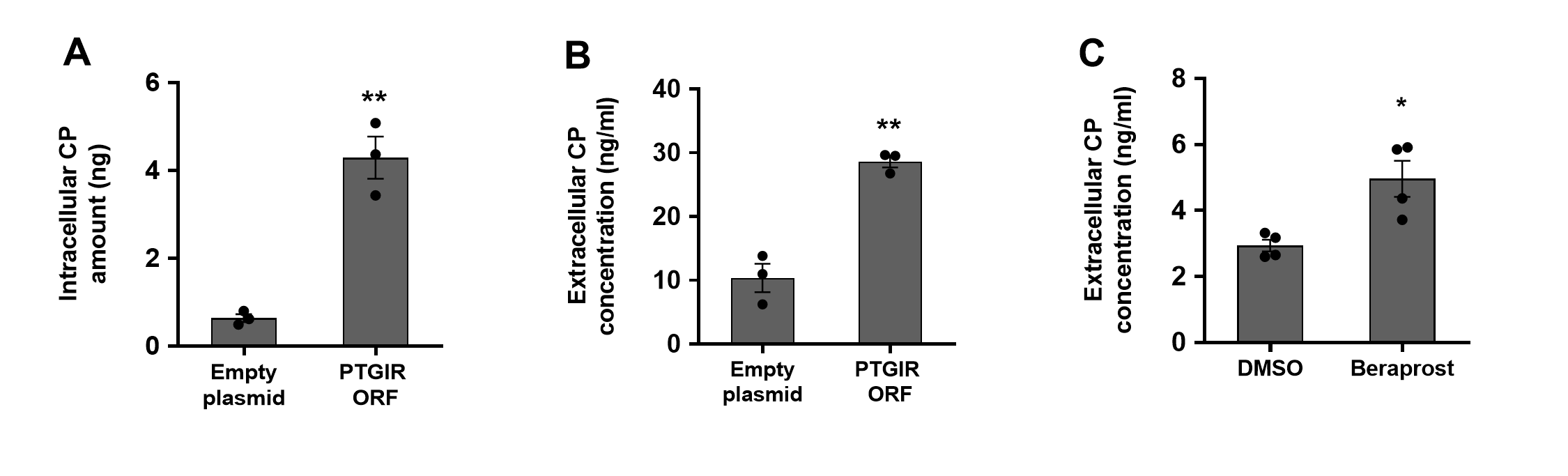

Supplement: S8 Fig — (TIF) [file pgen.1010189.s018.tif]

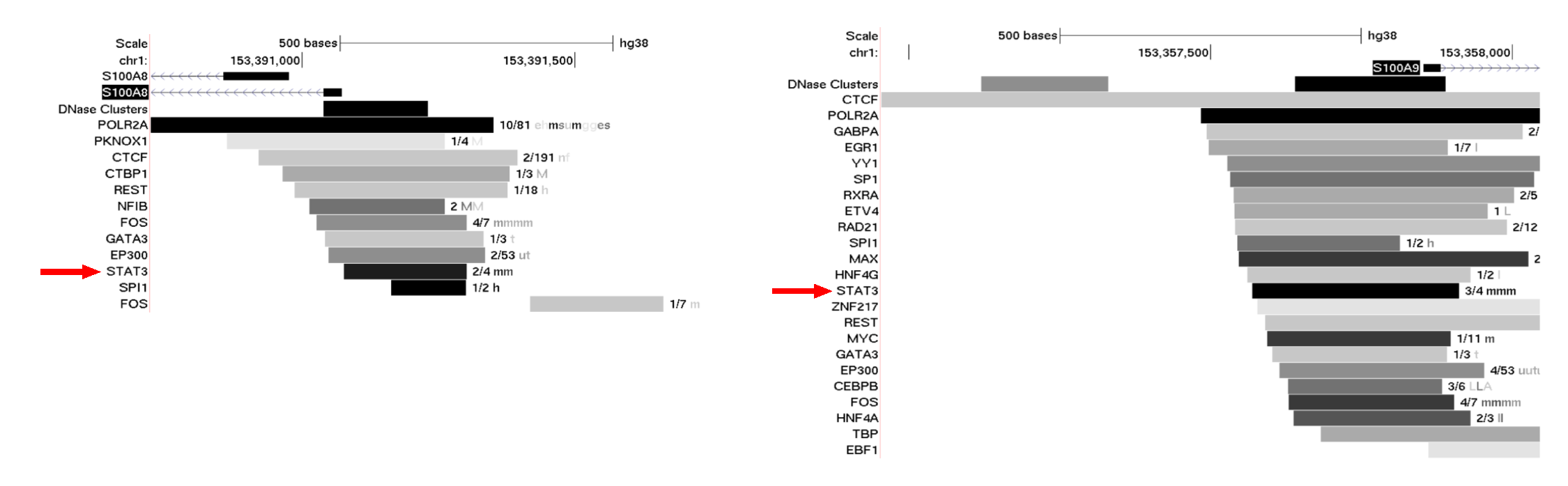

Supplement: S9 Fig — (TIF) [file pgen.1010189.s019.tif]

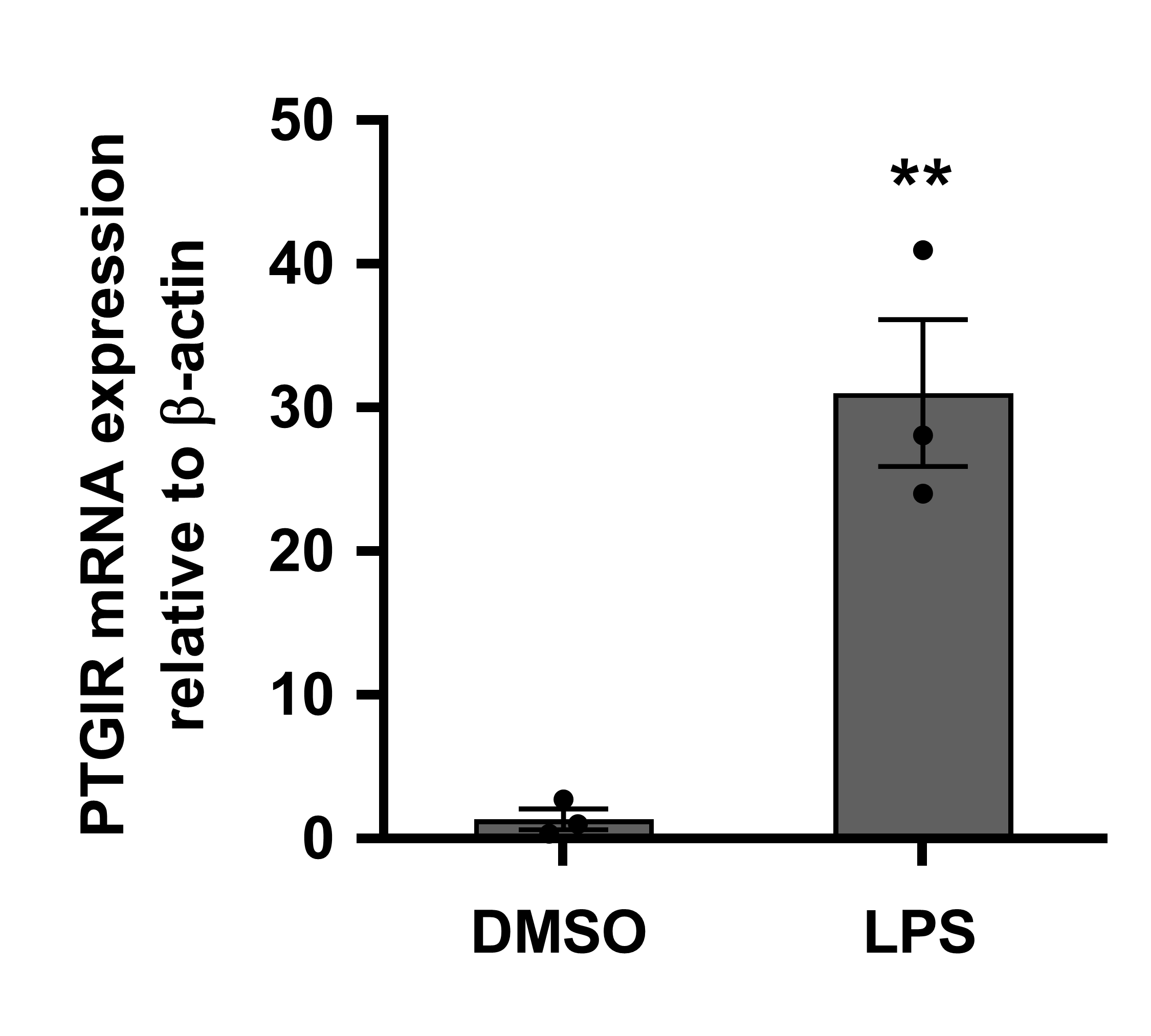

Supplement: S10 Fig — (TIF) [file pgen.1010189.s020.tif]

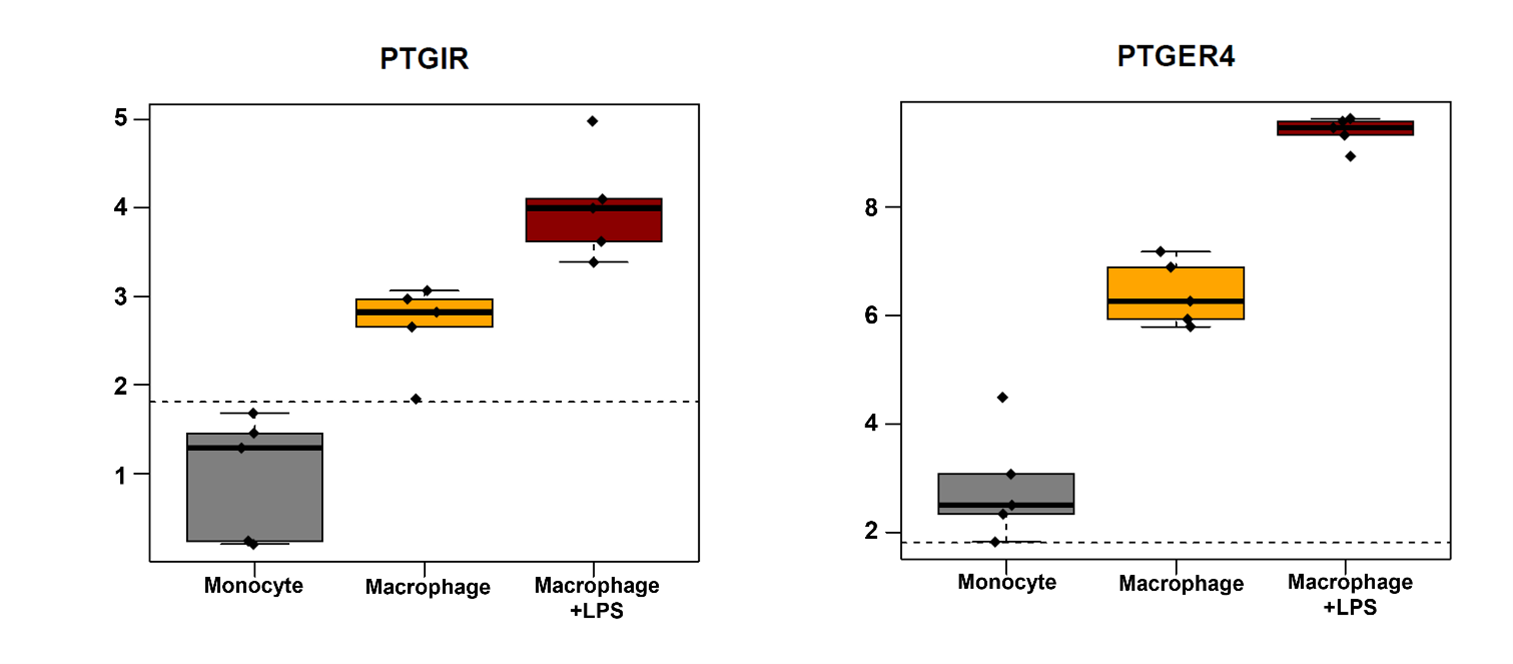

Supplement: S11 Fig — (TIF) [file pgen.1010189.s021.tif]

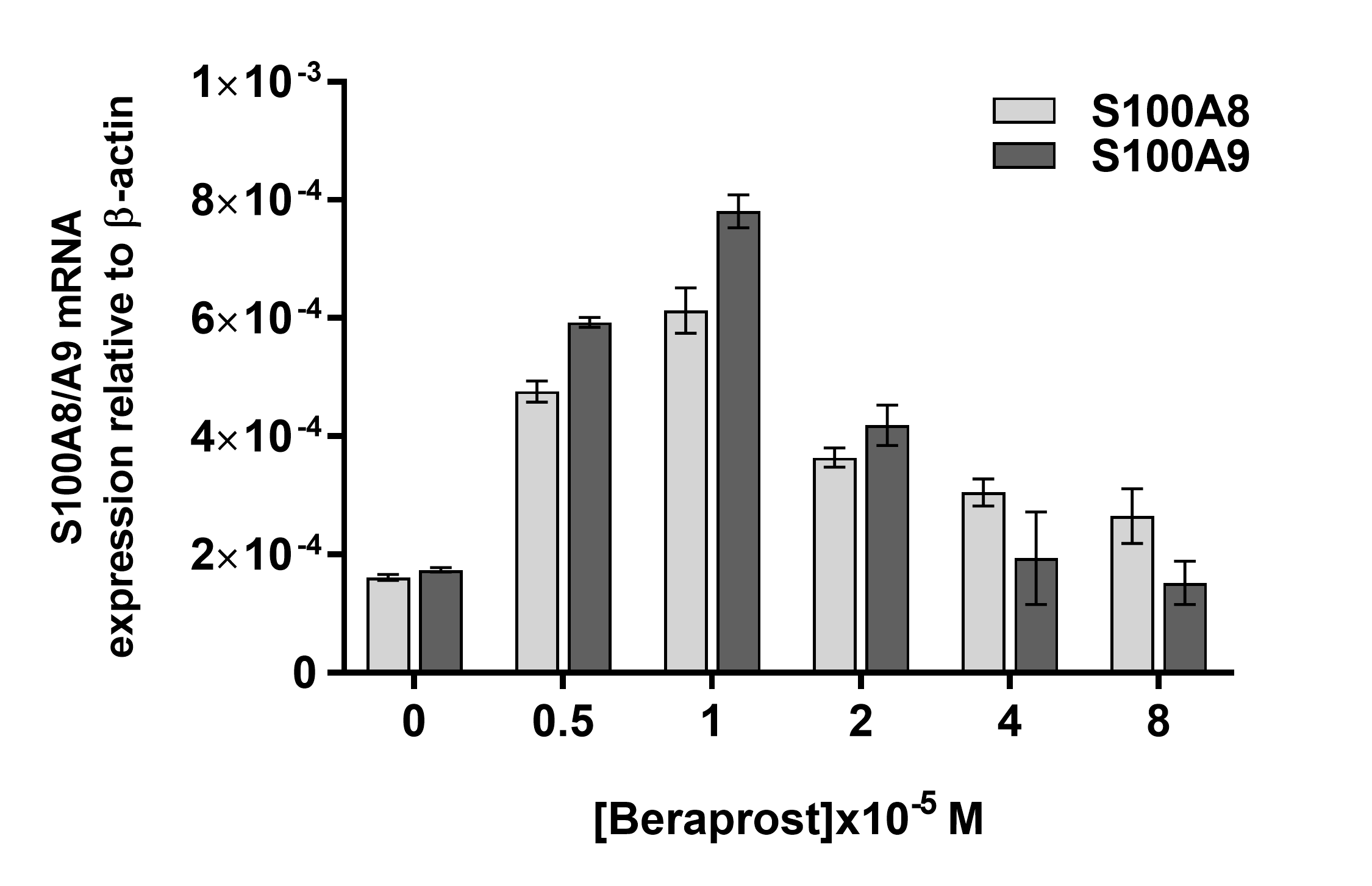

Supplement: S12 Fig — (TIF) [file pgen.1010189.s022.tif]

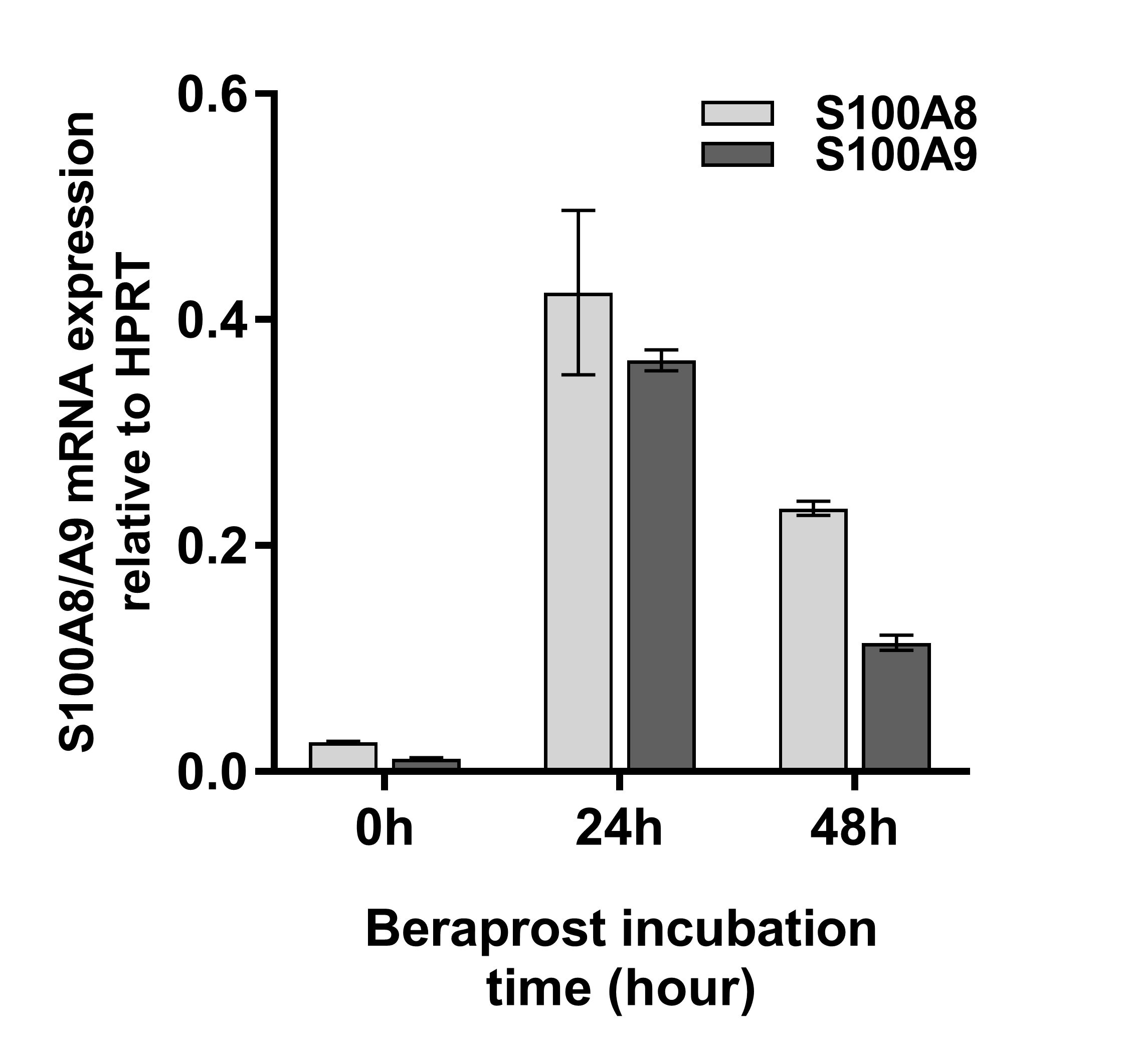

Supplement: S13 Fig — (TIF) [file pgen.1010189.s023.tif]

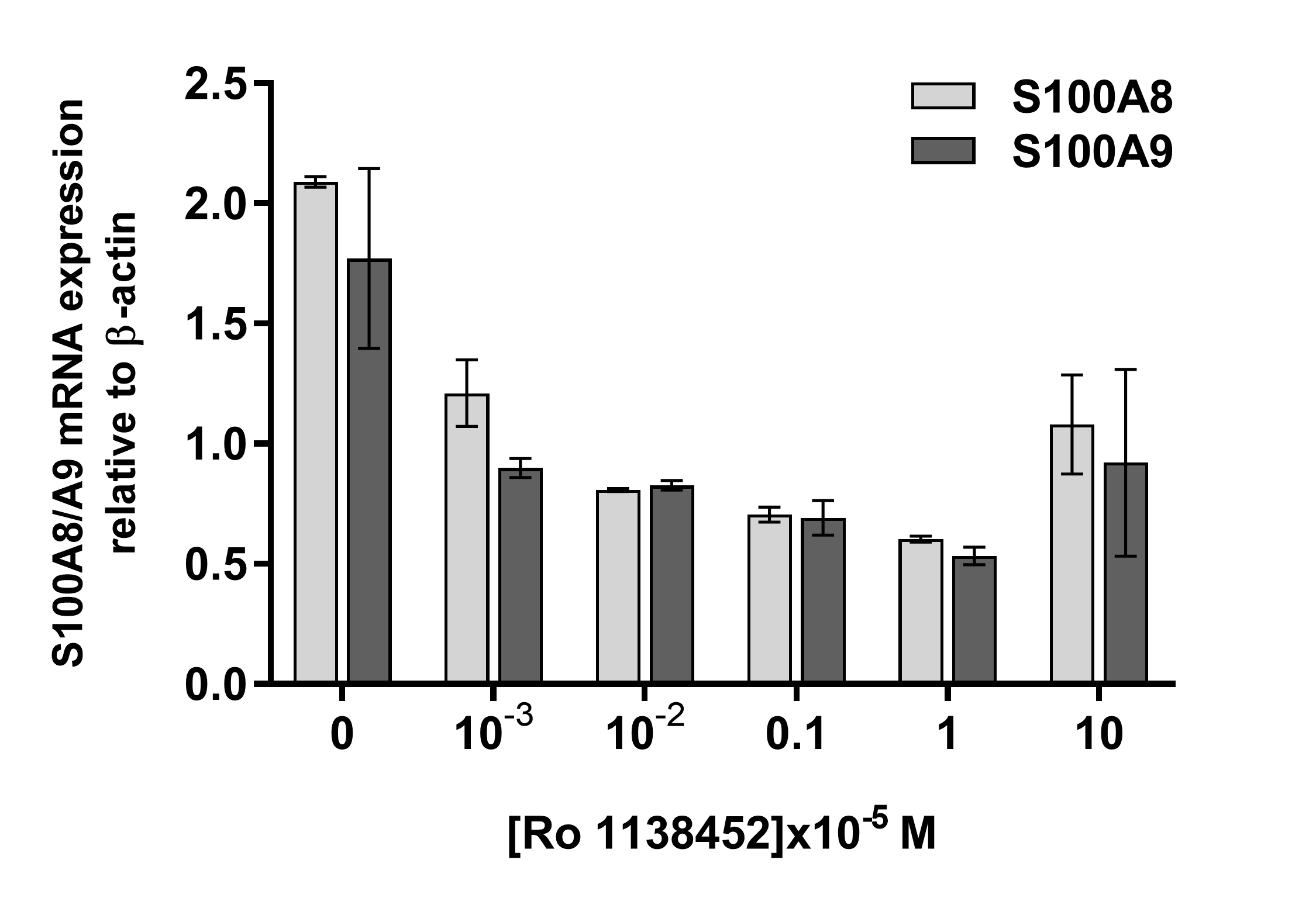

Supplement: S14 Fig — (TIF) [file pgen.1010189.s024.tif]
