# Supplementary material for: Prostaglandins and calprotectin are genetically and functionally linked to the Inflammatory Bowel Diseases
Source: PLoS Genet. 2022 Sep 26;18(9):e1010189. doi: 10.1371/journal.pgen.1010189 (PMC9536535; doi:10.1371/journal.pgen.1010189)
Supplement: S1 Appendix — Graphs illustrating the impact observed on the transcriptome of THP-1 cells following the expression of each ORF (42 ORFS). In the first four (ZBTB40, SLC39A11, NFKB1, PTGIR), the S100A8 and S100A9 genes are labeled. For the description of graphs see Methods section. (PDF) [file pgen.1010189.s025.pdf]

Supplementary graphs

Impact of the expression of all 43 IBD gene candidate ORFs on the transcriptome of THP-1 cells. Graphs illustrating the impact observed on the transcriptome of THP-1 cells following the expression of each ORF (43 ORFS). In the first four (ZBTB40, SLC39A11, NFKB1, PTGIR), the S100A8 and S100A9 genes are labeled. For the description of graphs see *Methods section.*

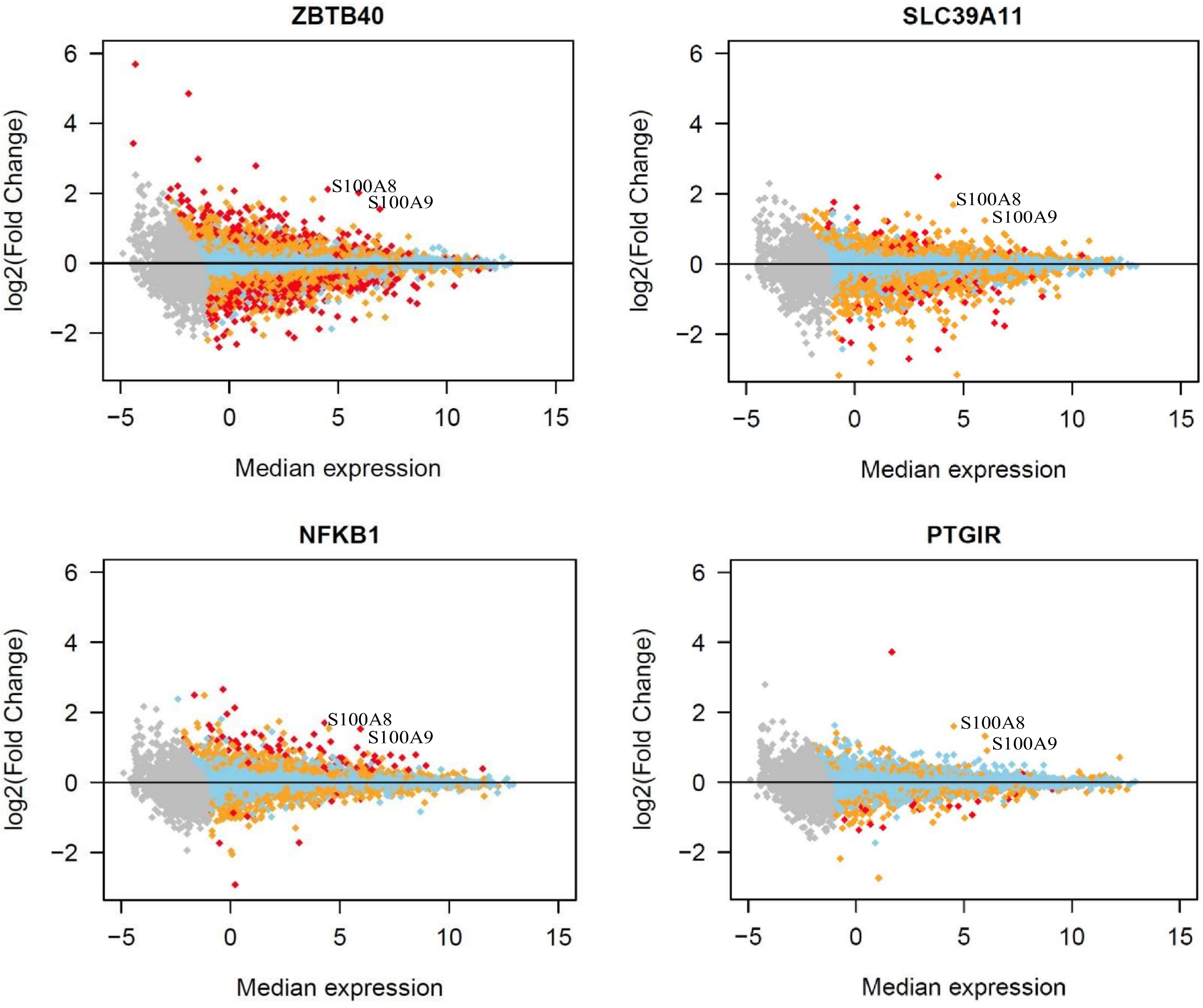

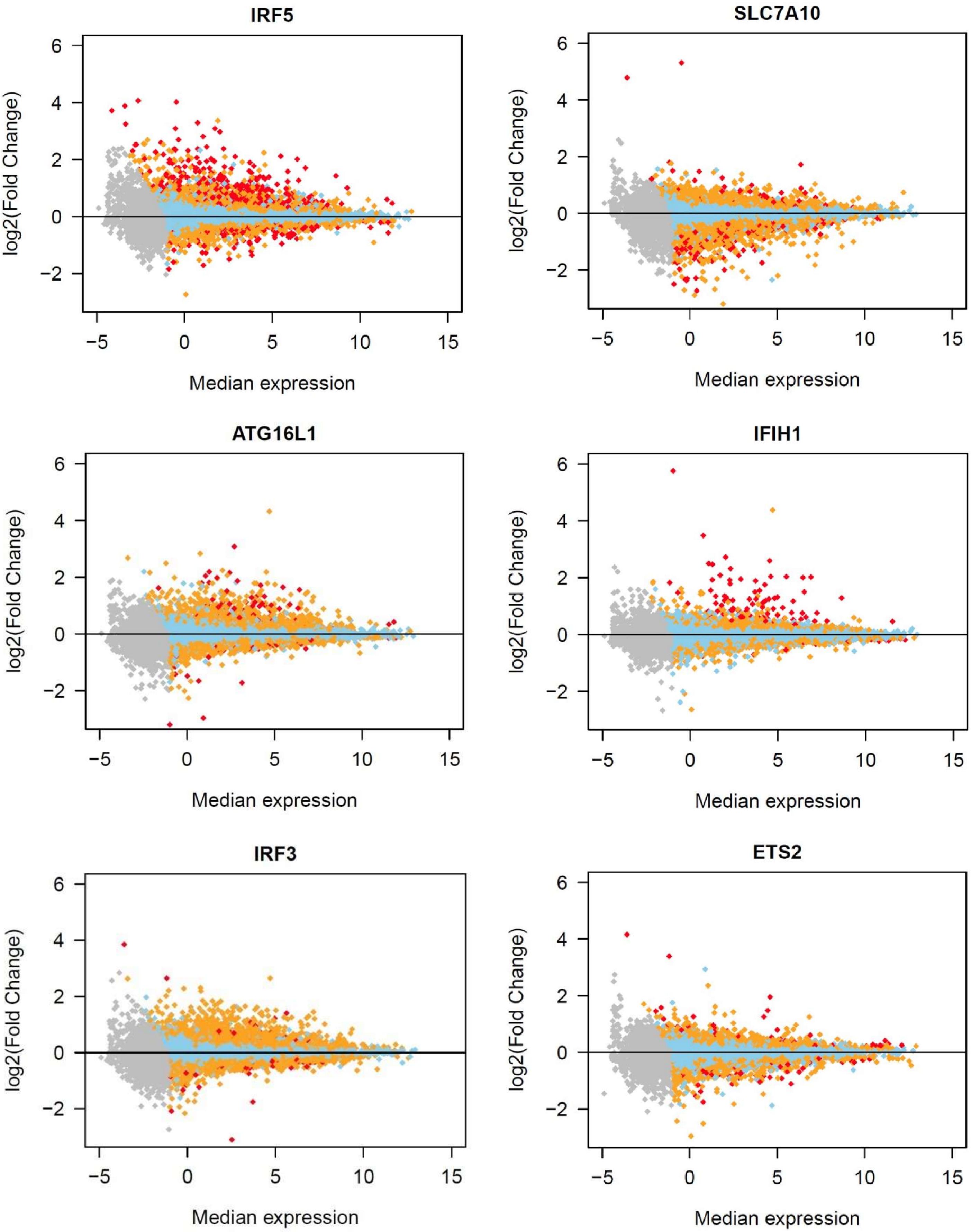

IRF5
SLC7A10
ATG16L1
IFIH1
IRF3
ETS2
log2(Fold Change)
6
4
2
0
−2
−5
0
5
10
15
Median expression

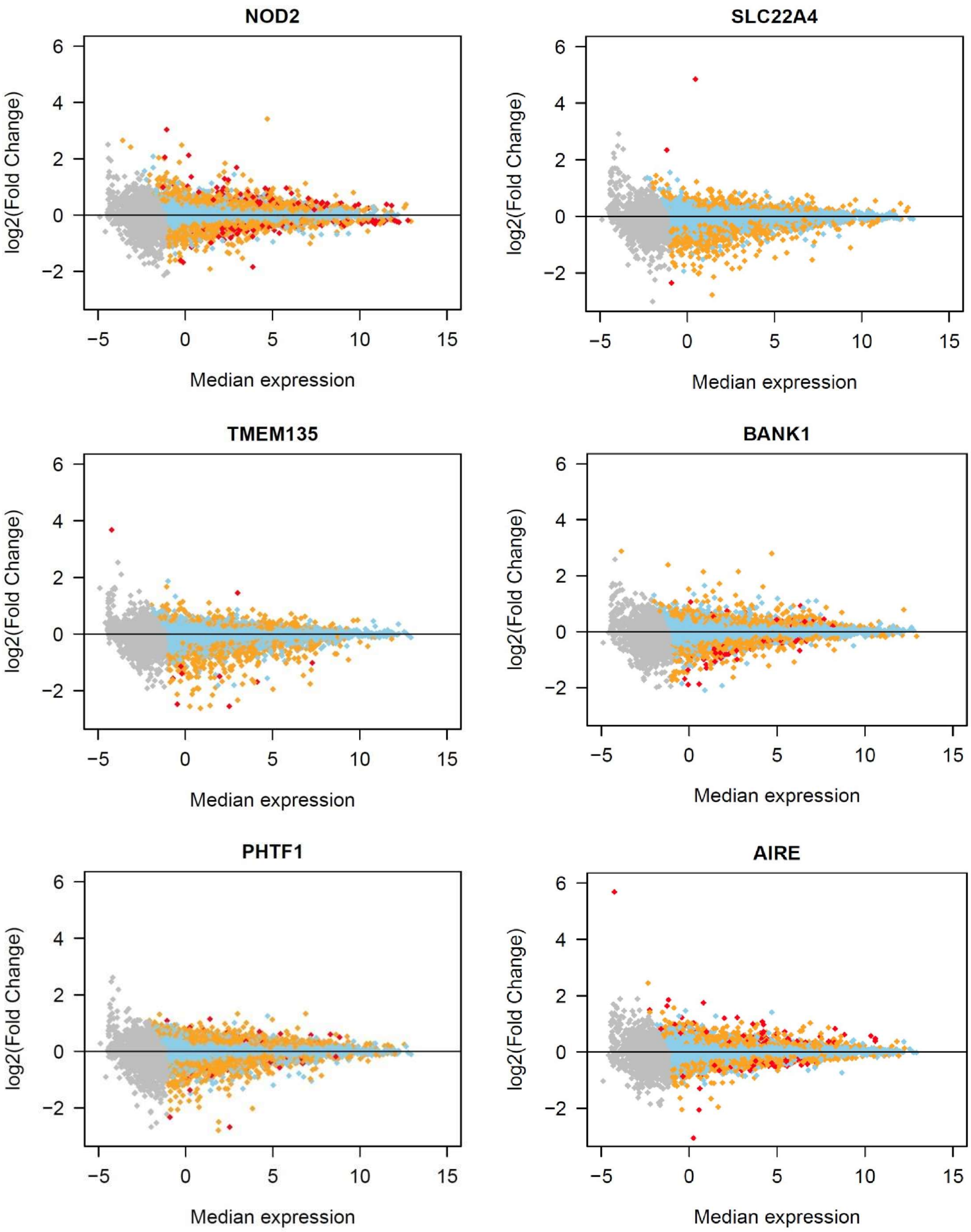
NOD2
SLC22A4
TMEM135
BANK1
PHTF1
AIRE
log2(Fold Change)
Median expression
6
4
2
0
−2
−5
0
5
10
15

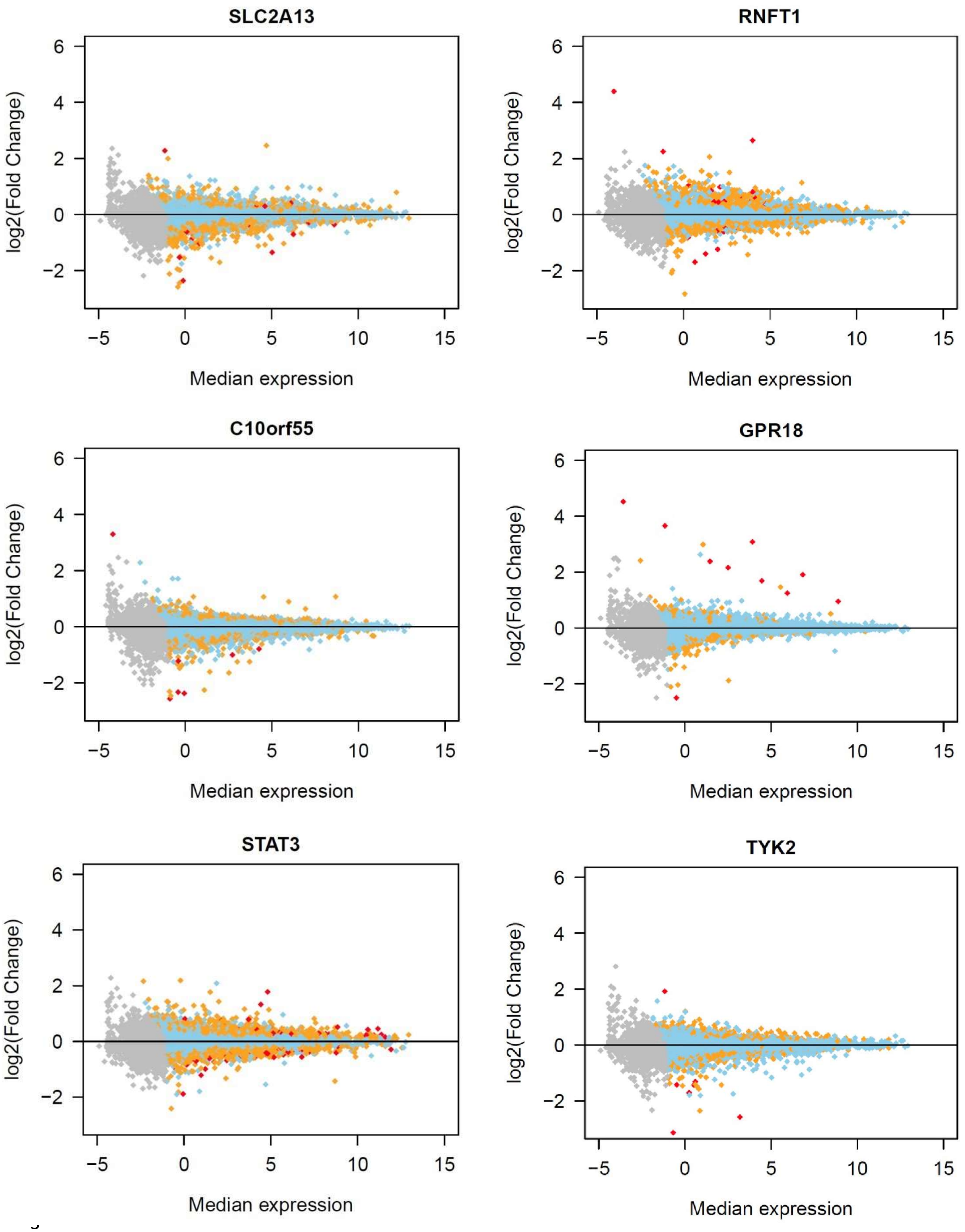
SLC2A13
RNFT1
C10orf55
GPR18
STAT3
TYK2
log2(Fold Change)
Median expression
6
4
2
0
−2
−5
0
5
10
15

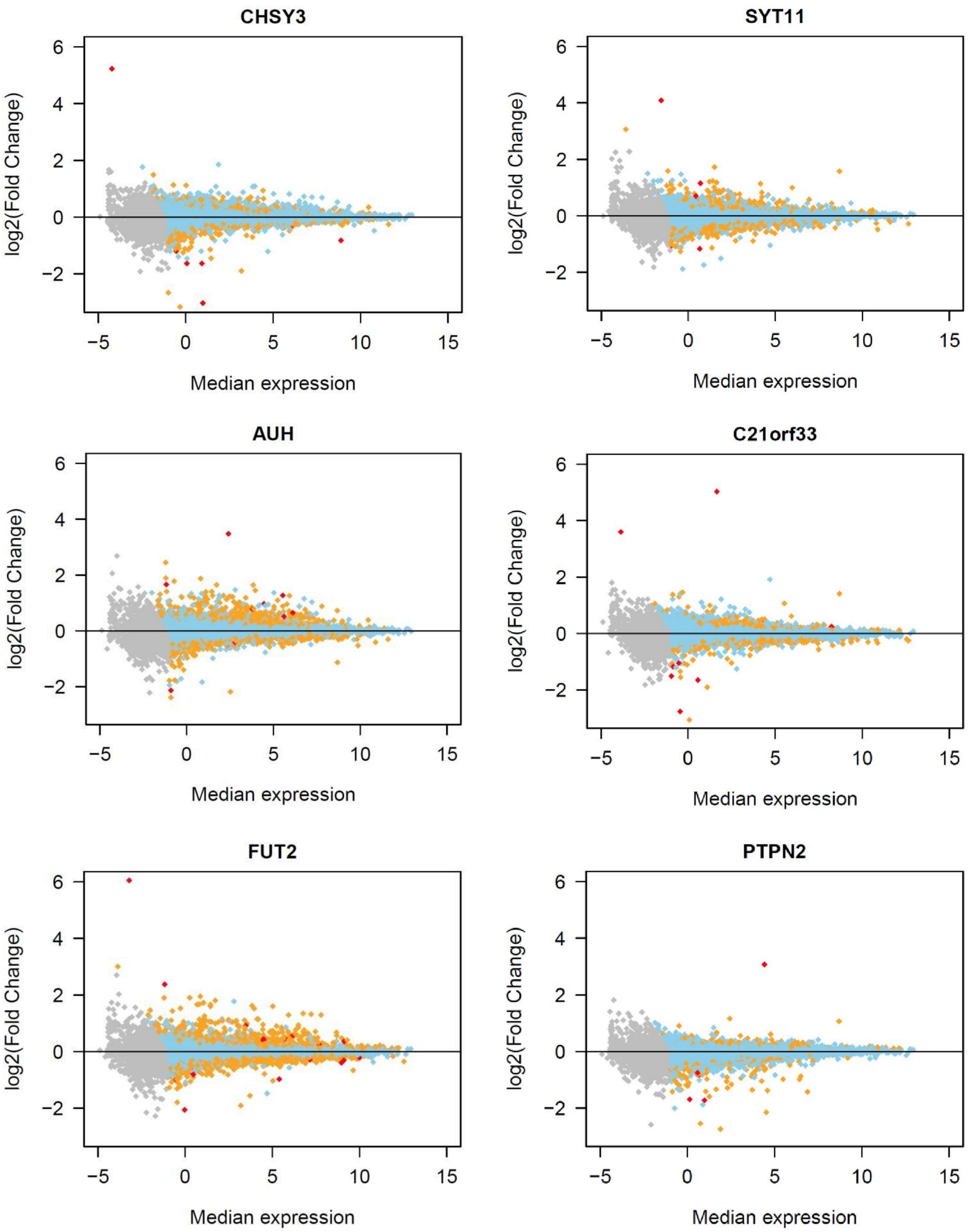

CHSY3
SYT11
AUH
C21orf33
FUT2
PTPN2
log2(Fold Change)
Median expression
6
4
2
0
−2
−5
0
5
10
15

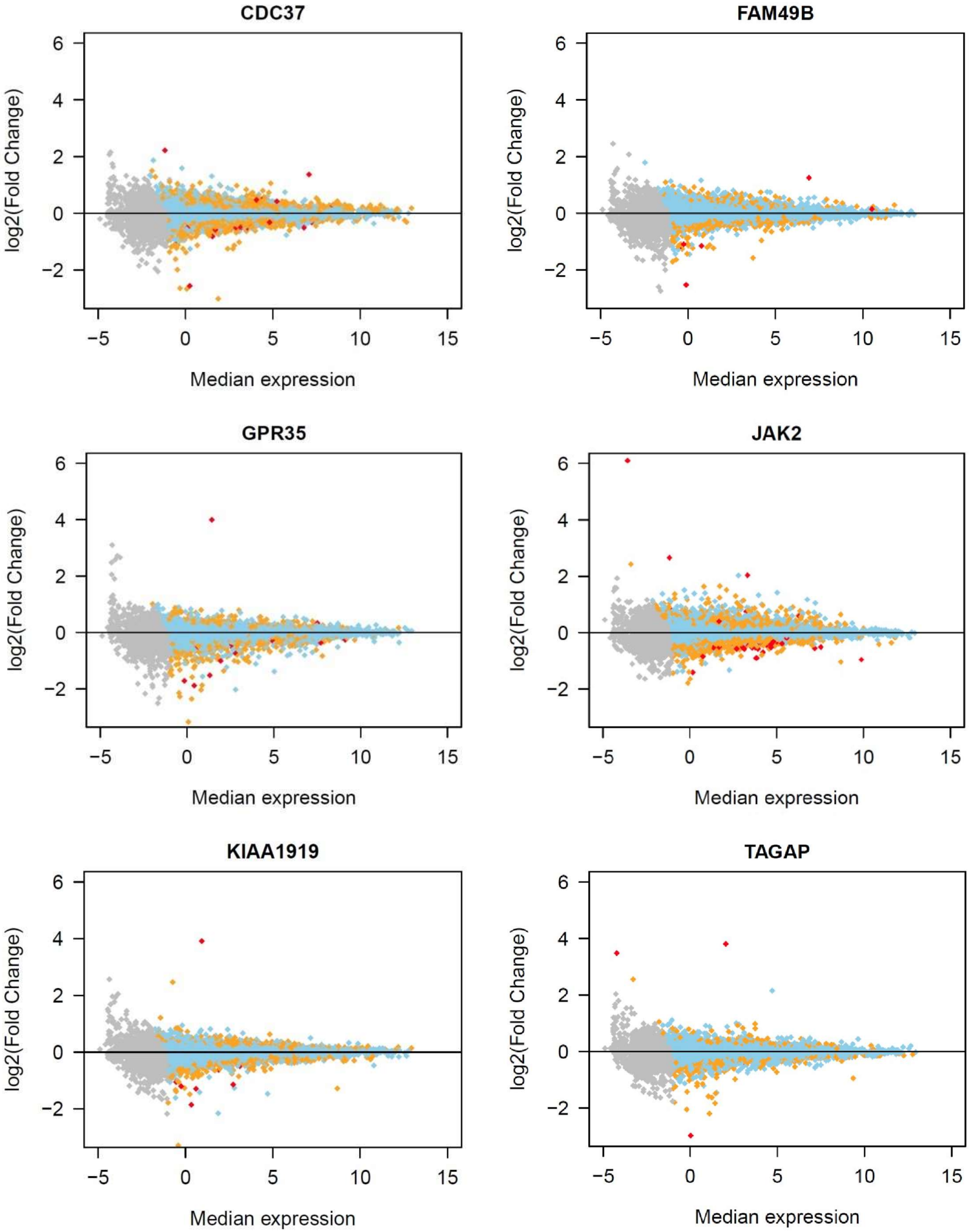

CDC37
FAM49B
GPR35
JAK2
KIAA1919
TAGAP
log2(Fold Change)
Median expression

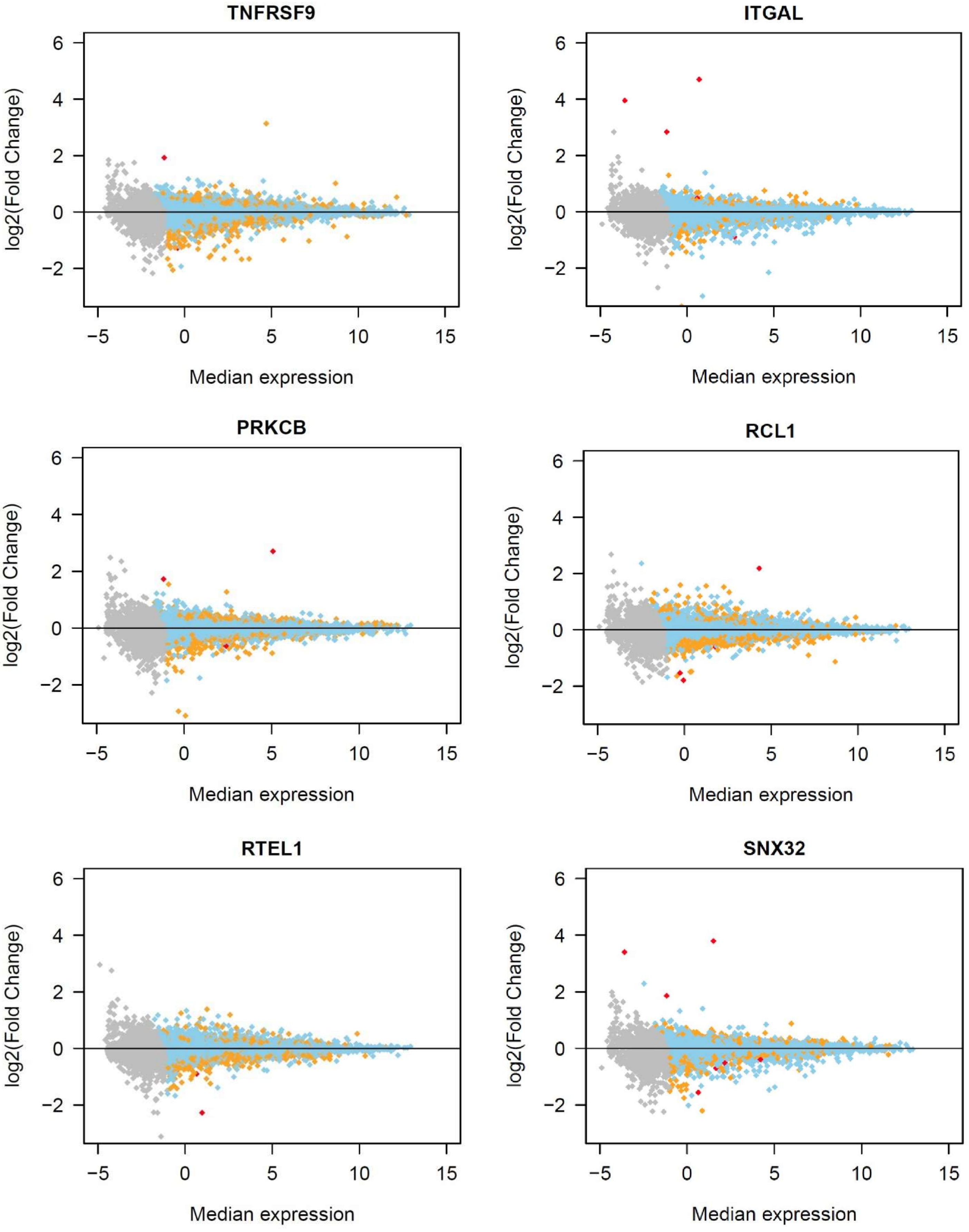
TNFRSF9
ITGAL
PRKCB
RCL1
RTEL1
SNX32
log2(Fold Change)
Median expression
6
4
2
0
−2
−5
0
5
10
15

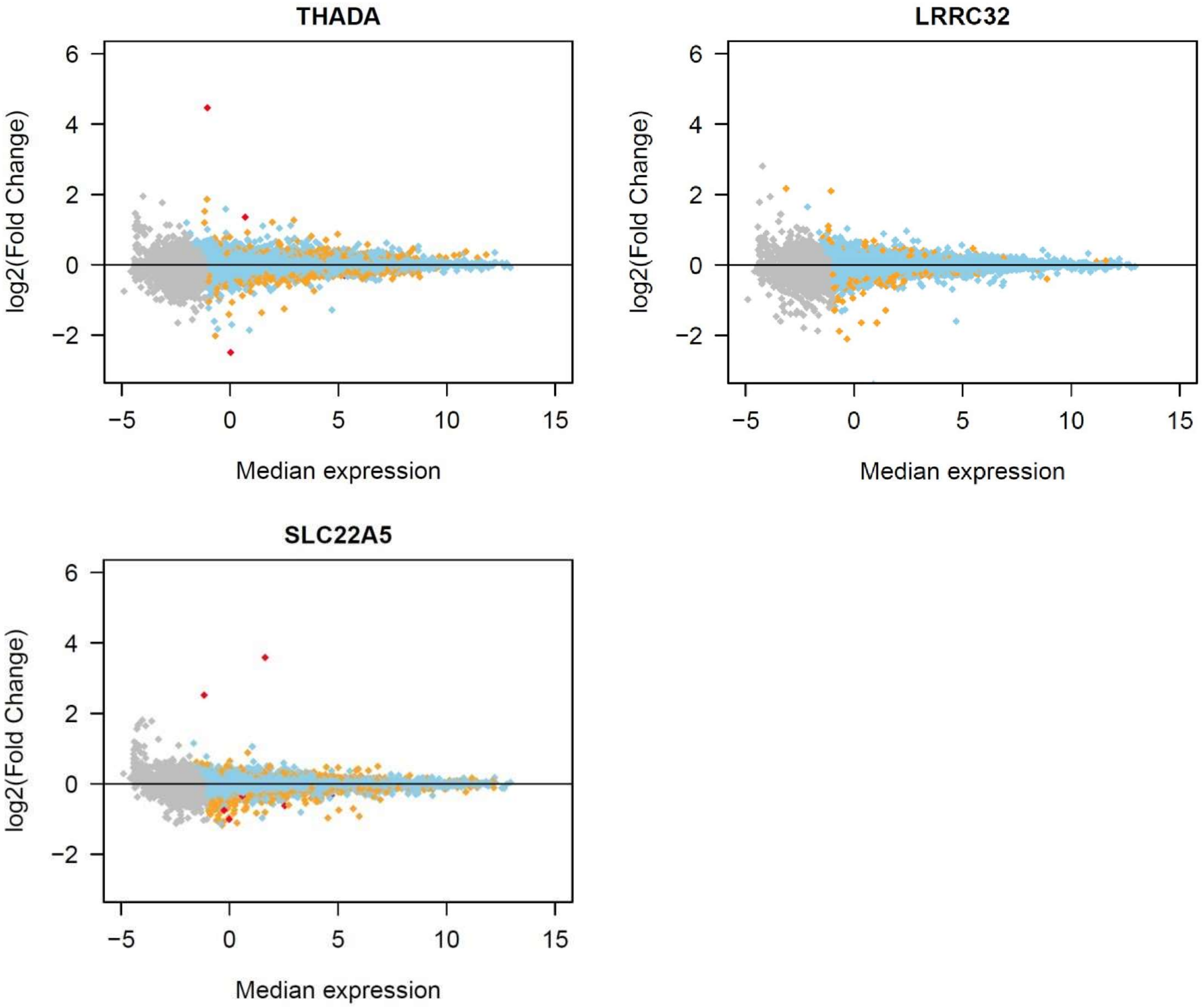
THADA
LRRC32
SLC22A5
log2(Fold Change)
Median expression
6
4
2
0
−2
−5
0
5
10
15
