# Supplementary material for: Prostaglandins and calprotectin are genetically and functionally linked to the Inflammatory Bowel Diseases
Source: PLoS Genet. 2022 Sep 26;18(9):e1010189. doi: 10.1371/journal.pgen.1010189 (PMC9536535; doi:10.1371/journal.pgen.1010189)
Supplement: S1 Methods — (DOCX) [file pgen.1010189.s026.docx]

**Supplementary Methods**

### Expression profiling of IBD gene candidates

### Expression profiling of all the genes located in the IBD 163 loci (297 genes) was performed across a panel of different RNAs from human tissues (n = 1, purchased from Clontech Laboratories), isolated human primary circulating immune cells (n=6 for each), and from immortalized intestinal and immune cell lines (n = 3, cell line from ATCC). RNA was isolated using RNeasy Plus Mini kit (Qiagen) according to the manufacturer's protocol and using Agilent iGenex v.2 gene expression array. The expression level for each gene in each replicate has been calculated using the geometric mean of all probes within the gene, then the median was normalized across all genes in the array. The geometric mean was calculated for each tissue from at least 3 independent measurements. Only one sample was available for tissues, so we used technical replicates. A minimal expression value of 8 (or 3 in the log2 transformed data) was used as a threshold to identify expressed genes.

**Cloning of IBD Open Reading Frames (IBD-ORFs)**

First, we queried the Ultimate_TM_ ORF Lite Clone Collection library (Invitrogen) for the presence of pEntry plasmid clones containing the longest validated isoform of each ORF, as defined by the NCBI’s Consensus CDS (CCDS) project. The cloned ORF sequences (supplied with the library) were then screened for point mutations affecting the primary amino acid sequence of the protein. In the cases where a SNP was found affecting the amino acid sequence, we only retained the cloned ORF if it contained the common allele. For any ORF either absent from the library, showing only smaller isoforms, or carrying changes to the accepted primary sequence of the protein, a full-length codon-optimized attB-flanked sequence was synthesized using Invitrogen GeneArt gene synthesis (Invitrogen) and inserted into pDONR vector using BP reaction. All ORFs were then cloned into the pLVX-EF1a-IRES-PURO/eGFP vector using LR reaction, downstream of the constitutive promoter and upstream of an IRES-controlled Puromycin-GFP hybrid reporter gene, so that transfection, transduction and expression could be monitored by fluorescence. Cloning was performed using the GATEWAY® cloning system, and cloned ORFs were fully sequenced to rule out any point mutations or cDNA rearrangements impacting on the primary protein sequence.

**Production of lentiviral stocks**

Lentiviral particle stocks were produced for all cloned IBD gene candidate ORFs. Lentiviral expression vectors were purified with GenElute HP Plasmid Maxiprep Kit (Sigma, St-Louis, MO) and only plasmid preparations with absorbance (260/280) ratios 1.8 to 2.0 were used for transfection. All ORFs cloned into the pLVX-EF1a-IRES-PURO/eGFP lentiviral expression vector were added to lentiviral packaging and envelope vectors (Sigma-MISSION-gag-pol and Sigma-MISSION-VSV-G respectively (Sigma)) in a 2:2:1 ratio and co-transfected into HEK-293T packaging cells via the calcium phosphate precipitation method according to the Open-Biosystem protocol (as described in (2)). Following an incubation of 7-8 hours in DMEM supplemented with 10 % FBS, 1% L-Glutamine 2 mM, 0.1% Pen-Strep at 37°C under 5% CO_2,_ the culture media was replaced with fresh culture media and the cells were incubated for an additional 40 hours. Lentivirus-containing medium was harvested, cell debris pelleted, and viral particles were concentrated using the Lenti-X Concentrator (Takara Bio), resuspended with McCoy’s 5A (Wisent) in 1/10 of the original volume (10X concentration). The lentiviral stock was divided in 100 ul single-use aliquots and stored at -80°C.

**Lentiviral transduction and antibiotic selection in THP-1 cells**

Briefly, 4x10^5^ cells were centrifuged at 2500 RPM for 90 min at 32 °C with ORF-specific lentivirus (MOI of 0.05-0.5) and 8µg/ml of polybrene (Sigma) to improve transduction efficiency. Immediately, after centrifuging the medium containing the virus was discarded and the pellet resuspended in 2ml of complete medium in 6 well plate (Corning-Thermo Fisher Scientific). Seventy-two hours post-transduction, media was changed and 1ug/ml puromycin (Millipore Sigma) was added. The selective medium was changed every 2 days until the non-transduced negative control cells had completely died. Cell survival was assessed by trypan blue staining every 2 days. Following selection, the concentration of puromycin was reduced to 0.2 µg/m to maintain the stable lines. The appearance and density of the cultures were recorded daily, and cells were grown for an average of 7 days (with a range of 4-16 days) to select successfully transduced cells and reach 1x10^6^ cells/ml (3-5 x10^6^ cells in total) before RNA extraction.

### Transcriptome and bioinformatics analysis

### Raw FASTQ formatted sequence files were retrieved from the McGill/Genome-Québec Innovation Center sequence service. Primary QC analysis was performed using FastQC (v0.11.5; <https://www.bioinformatics.babraham.ac.uk/projects/fastqc>). This step allowed us to set sequence filtering parameters to be used with Trimmomatic (v.0.36 )(3) Primer sequences were filtered out with provided TruSeq sequence data and a quality sliding window of *Q* over 20 for a 40-nucleotide window; only sequences longer than 70 nucleotides were kept in the filtered FASTQ files. Another FastQC run was performed on all filtered FASTQ files to validate Trimmomatic results.

Filtered FASTQ files were aligned to the human genome with STAR (v. 2.5.3a)(4) using indexes built with STAR from NCBI GRCh38 data (reference genome and GTF formatted annotation files ) downloaded from Illumina iGenome website. Default values were selected except for the –quantMode flag that was set to TranscriptomeSAM for downstream quantification. Counts per gene for each sequenced sample were obtained using RSEM (v. 1.2.6)**(5)**, building its own indexes using the same NCBI GRCh38 data files as STAR and read into R for statistical analysis (<https://www.r-project.org>).

Gene counts were transformed to FPKM as an approximation of gene relative abundance. Genes were filtered to keep only those with FPKM > 0.3 and count>8 in at least two samples. Small RNAs and LOC genes without annotations were removed. Quality control of samples was performed, excluding strong outlier samples based on principal component analyses. The log-transformed FPKM were then normalized between samples with cyclicloess (normalizeCyclicloess from sva Bioconductor R package). RNA-seq and cell culture batches effects were removed using ComBat (sva Bioconductor library v3.18.0).

**HIT definition**

HITS are identified as genes with detectable expression in THP-1 (either endogenously or following ORF expression) for which the fold effect (increase or decrease) in response to the expression of a given ORF is greater than two compared to the baseline obtained from all samples tested and shows expression outside the expected range.

More precisely, we expect expression of the different ORFs to have distinct effects on the expression pattern of different genes in the transcriptome, we leveraged the information from the complete dataset to generate the baseline distribution of expression for all genes expressed in the THP-1 cell line under the culture conditions used in this project. After log_2_ transformation of the expression data, ­the median expression measured for each gene over all samples was used as the baseline, while the median absolute deviation (MAD) was used to define expected range of variability. This information was summarized as a Z score computed as: $Z=(\bar{x}-b)\frac{\sqrt{n}}{MAD}$, where *n* is the number of replicates (*n*=3), $\bar{x}$ is the average from the replicates and $(\bar{x}-b)$ is the difference from the baseline. Genes with |Z| > 4 were considered outside expected range of variation. For each expressed ORF, a gene was defined as a HIT if the deviation $(\bar{x}-b)$ was larger than 1, which is equivalent to a fold effect greater than 2 on the original scale, and the expression was outside the expected range of variation (|Z| > 4).

**Enrichment analyses**

In order to find biological categories enriched in HITS identified in the screen, we performed enrichment analyses using the g:GOSt functional profiling tool (using Ensembl database release 98) from the online g:Profiler service (<https://biit.cs.ut.ee/gprofiler/gost>) (6) Specifically, we evaluated enrichment for Gene Ontology (GO) terms (Biological process (BP), molecular function (MF) and cellular compartment (CC); release 2020-06-02), biological pathways (KEGG, release 2020-06-01; Reactome, release 2020-06-11; and WikiPathways, release 2020-07-06), regulatory motifs in DNA (TRANSFAC, release 2020.1) and Protein databases (CORUM, comprehensive resource of mammalian protein complexes; release 2018-09-03) enrichment with a corrected *P*<.05 (using the g:SCS algorithm intrinsic to gProfiler which takes into account hierarchically related terms) were considered.

In terms of cis-regulatory motif analyses of the proximal promoters of HITS identified in the screen we also used the PRIMA method (7) as implemented in the EXPANDER software (v8.0)(8). Specifically, we performed enrichment analyses aimed at detecting cis-regulatory DNA motifs (Jasper database) that were over-represented in the promoter sequences (Ensembl database v89 built on 2020-03-07) of the HITS for each of the ORFs expressed in THP-1. Only enrichment of greater than 2-fold with a corrected *P*<.05 (using the FDR method) were considered.

**Lentiviral production for PTGIR knock-down**

Lentiviral stocks for PTGIR shRNA-expression vectors were produced following the same procedure described above for the ORFs. Five independent PTGIR-targeting shRNA lentiviral vectors PLKO-puro (TRCN0000014179, TRCN0000014180, TRCN0000014181, TRCN0000014182 and TRCN0000358057) and an empty plasmid with no shRNA were transduced in THP-1 cells via spinoculation. Three shRNA PTGIR knockdown and shRNA non-target cell lines were derived for each shRNA vector following selection with 1 ug/ml puromycin and were maintained at 37°C with 5% atmospheric CO2 in RPMI 1600 complete medium. Total RNA was extracted, and cDNA synthesized as described above. The level of PTGIR RNA expression was quantified by qPCR and the shRNA clone with the highest knock down efficiency (> 80%) on endogenous PTGIR expression levels (TRCN0000358057) was selected to use in subsequent experiments.

**Gene expression quantification**

Total RNA was extracted from cells and cDNA synthesized. 100ng of RNA from each sample was reverse transcribed into cDNA using the High-Capacity cDNA RT kit (Thermo Fisher Scientific) with nuclease inhibitors (Invitrogen, Thermo Fisher Scientific). The cDNA was amplified by qPCR using the PowerUp SYBR Green Master mix reagent according to the manufacturer's recommendations (Thermofisher) and with the QuantStudio 6 thermal Real time PCR using specific primers (**Table S9 in Supporting Tables**). Then, the RNA expression levels of genes PTGIR, ZBTB40, S100A8, S100A9, TNF were quantified by qPCR and normalized to mRNA expression levels of β-actin. RNA relative expression was calculated using 2^-ddCt. Statistical differences between mRNA expression levels of different groups were calculated by Student’s paired t-test.

**THP-1 response to different concentration and incubation time of PTGIR agonist and antagonist**

To determine the best concentration of PTGIR agonist, S100A8/A9 expression levels were measured with respect to different concentration of Beraprost sodium (0, 0.5, 1, 2, 4, 8) x10^-5^M (**S12 Fig**). A time course of S100A8/A9 mRNA expression induction by PTGIR agonist was also performed after treating THP-1 cells with Beraprost sodium for 24 and 48h (**S13 Fig**) to determine the optimal time for our experiments. A dose response of S100A8/A9 RNA expression to PTGIR antagonist Ro 1138452 (0, 10^-3^, 10^-2^, 0.1, 1, 10) x10^-5^M was performed in THP-1 cells transduced with the PTGIR ORF (**S14 Fig**).

**Measurement of CP protein by ELISA**

Supernatant and cell lysates were collected from THP-1 stable lines transduced by PTGIR ORF or by the empty vector. The calprotectin levels were detected in samples using Human S100A8/S100A9 Heterodimer DuoSet ELISA (R&D system) according to the manufacturer’s instruction.

**Lymphoblastoid cell lines (LCLs) reprogramming into hiPSCs**

Human lymphoblastoid cell lines (LCL) collected by the NIDDK IBD Genetics Consortium from healthy controls (HC) were obtained from the NIDDK Central Repository ([https://repository.niddk.nih.gov](https://repository.niddk.nih.gov/)). LCL reprogramming was performed as described by Kumar *et al.*(9). Briefly, 1x10^6^ cells were nucleofected with four episomal reprogramming plasmids (pCE-hUL, pCE-hSK, pCE-hOCT3/4, and pCE-mp53DD) using the Amaxa® Human Monocyte Nucleofector® Kit (Lonza) according to the manufacturer’s protocol. After an 8-12 hour recovery period in complete media (RPMI1640-GlutaMAX™ (Life Technologies) supplemented with 20% FBS (Sigma) and 1% P/S (Wisent)), the cells were transferred to Matrigel® (Corning) coated plates in TeSR™-E7™ (STEMCELL™ Technologies) reprogramming media. Cells were then cultivated for 13 to 15 days, and when hiPSC-like colonies started to appear, the growth media was changed to mTeSR™1 (STEMCELL Technologies). Colonies were tested for TRA-1-60 expression between days 20 and 22, and four TRA-1-60 colonies were picked and expanded for each cell lines. Each hiPSC cell line was then further sub-cloned by sorting of single cells into single wells of Matrigel®-coated 96-well plates containing Stemflex (Gibco^TM^) media supplemented with 1X Revitacell (Gibco^TM^) using a FACSAria Fusion (BD) cell sorter. Media was changed every 3-4 days and the amount of Revitacell was reduced to 1/2X after 6 days. Each well was investigated for the emergence of colonies derived from single cells and 2 sub-clones of each cell line were selected, characterized, and expanded for further studies after 11-12 days.

**hiPSC characterization**

Each hiPSC sub-clonal line was then tested for the absence of genomic integration of the four plasmids used for LCL-hiPSC reprogramming, via a PCR amplification of plasmid DNA using HotStarTaq Plus DNA Polymerase (Qiagen) (See **S9 Table** **in Supporting Tables** for the list of primers used). The hiPSC lines were also tested for the expression of pluripotency markers by RT-qPCR amplification of the genes NANOG, SOX2 and POU5F1 (**S9** **Table in Supporting Tables and S6 Fig** for the list of primers used). Finally, the capacity of each of these sub-clonal hiPSC lines to differentiate into the three germ layers was assessed using the Human Pluripotent Stem Cell Functional Identification Kit (R&D Systems) following manufacturer’s instructions.

**Differentiation of hiPSCs into monocytes**

The differentiation of hiPSC lines into monocytes was performed as described by Yanagimachi, M. D. *et al.*(10). Briefly **(S6 FigA)**, 30 colonies obtained using the ReLeSR™ solution were seeded in a 100mm Petri dish containing mTeSR1 medium. At Day 0, BMP4 (Sigma Aldrich, 80 ng/mL) was added to the mTeSR1 medium. Between Days 4 and 5, medium was changed to Stempro®-34 SFM (1X) (Gibco^TM^) supplemented with VEGF (Sigma Aldrich, 80 ng/mL), bFGF (Sigma Aldrich, 25 ng/ml), and SCF (Sigma Aldrich, 100 ng/mL) to promote the development of hemoangiogenic progenitors. On Days 6 and 7, the cytokine cocktail was changed to SCF (50 ng/mL), IL-3 (R&D systems, 50 ng/mL), TPO (Thrombopoietin) (Sigma Aldrich, 5 ng/mL), M-CSF (Sigma Aldrich, 50 ng/mL), and Flt-3 ligand (Sigma Aldrich, 50 ng/mL) for the generation of hematopoietic cells. Finally, the cytokine cocktail was changed to Flt-3 ligand (50 ng/mL), GM-CSF (Sigma Aldrich, 25 ng/mL), and M-CSF (50 ng/mL) between Days 13 and 15 to drive the monocytic lineage-directed differentiation. Following that, free floating monocytes were harvested from the supernatant and the medium was changed every 3-4 days.

**Flow cytometry, immunophenotyping**

For immunophenotyping **(S6 Fig),** a minimum of 250,000 cells were harvested from the supernatant of monocyte differentiation cultures and washed in cold sorting buffer. Cells were blocked by incubating 20 µL of FcR Blocking Reagent (MACS Miltenyi Biotec) for 10 min at 4°C prior to antibody staining. For compensation settings, the Anti-Mouse Ig, κ/Negative Control Compensation Plus Particles Set (BD™ CompBeads Plus) was used according to the manufacturer’s protocol. The antibodies used were the following: BV421 Mouse Anti-Human TRA-1-60 (Clone TRA-1-60), APC Mouse Anti-Human CD14 (Clone M5E2), BB515 Mouse Anti-Human CD45 (Clone HI30) (BD Biosciences). Cells and beads were incubated with the antibodies 30 min at 4°C and then washed twice with cold sorting buffer. Cells were filtered using 70µM Pre-Separation Filters (MACS Miltenyi Biotec). Controls were made with the appropriated isotype controls. Stainings were acquired on a FACS Aria Fusion, and analyzed using FlowJo™ software.

**CD14+ cells enrichment with MACS**

For CD14+ cell enrichment, cells harvested from the supernatant of monocyte differentiation cultures were washed in cold sorting buffer and incubated with 20ul CD14 MicroBeads (MACS Miltenyi Biotec) for 15 min at 4°C. The cells were washed and placed on an LS column on a QuadroMACS^TM^ separator. CD14+ cells were retained in the column and flow through was discarded. The column was then removed from the QuadroMACS^TM^ separator and CD14+ cells were collected in cold sorting buffer.

**References**

1. Johnson AD, Handsaker RE, Pulit SL, Nizzari MM, O'Donnell CJ, de Bakker PI. SNAP: a web-based tool for identification and annotation of proxy SNPs using HapMap. Bioinformatics. 2008;24(24):2938-9.

2. Graham FL, van der Eb AJ. A new technique for the assay of infectivity of human adenovirus 5 DNA. Virology. 1973;52(2):456-67.

3. Bolger AM, Lohse M, Usadel B. Trimmomatic: a flexible trimmer for Illumina sequence data. Bioinformatics. 2014;30(15):2114-20.

4. Dobin A, Davis CA, Schlesinger F, Drenkow J, Zaleski C, Jha S, et al. STAR: ultrafast universal RNA-seq aligner. Bioinformatics. 2013;29(1):15-21.

5. Li B, Dewey CN. RSEM: accurate transcript quantification from RNA-Seq data with or without a reference genome. BMC Bioinformatics. 2011;12:323.

6. Raudvere U, Kolberg L, Kuzmin I, Arak T, Adler P, Peterson H, et al. g:Profiler: a web server for functional enrichment analysis and conversions of gene lists (2019 update). Nucleic Acids Res. 2019;47(W1):W191-W8.

7. Elkon R, Linhart C, Sharan R, Shamir R, Shiloh Y. Genome-wide in silico identification of transcriptional regulators controlling the cell cycle in human cells. Genome Res. 2003;13(5):773-80.

8. Hait TA, Maron-Katz A, Sagir D, Amar D, Ulitsky I, Linhart C, et al. The EXPANDER Integrated Platform for Transcriptome Analysis. J Mol Biol. 2019;431(13):2398-406.

9. Kumar S, Curran JE, Glahn DC, Blangero J. Utility of Lymphoblastoid Cell Lines for Induced Pluripotent Stem Cell Generation. Stem Cells Int. 2016;2016:2349261.

10. Yanagimachi MD, Niwa A, Tanaka T, Honda-Ozaki F, Nishimoto S, Murata Y, et al. Robust and highly-efficient differentiation of functional monocytic cells from human pluripotent stem cells under serum- and feeder cell-free conditions. PLoS One. 2013;8(4):e59243.

11. Takeda A, Hamano S, Yamanaka A, Hanada T, Ishibashi T, Mak TW, et al. Cutting edge: role of IL-27/WSX-1 signaling for induction of T-bet through activation of STAT1 during initial Th1 commitment. J Immunol. 2003;170(10):4886-90.

12. Kamiya S, Owaki T, Morishima N, Fukai F, Mizuguchi J, Yoshimoto T. An indispensable role for STAT1 in IL-27-induced T-bet expression but not proliferation of naive CD4+ T cells. J Immunol. 2004;173(6):3871-7.
